# Use of standardised patients to assess gender differences in quality of tuberculosis care in urban India: a two-city, cross-sectional study

**DOI:** 10.1016/S2214-109X(19)30031-2

**Published:** 2019-03-27

**Authors:** Benjamin Daniels, Ada Kwan, Srinath Satyanarayana, Ramnath Subbaraman, Ranendra K Das, Veena Das, Jishnu Das, Madhukar Pai

**Affiliations:** a Development Research Group, The World Bank, Washington, DC, USA; b University of California at Berkeley, Berkeley, CA, USA; c Center for Operational Research, International Union Against TB and Lung Diseases, Paris, France; d Department of Public Health and Community Medicine, Tufts University School of Medicine, Boston, MA, USA; e Institute for Socio-Economic Research on Development and Democracy, Delhi, India; f Department of Anthropology, Johns Hopkins University, Baltimore, MD, USA; g Center for Policy Research, New Delhi, India; h McGill International TB Centre and Department of Epidemiology and Biostatistics, McGill University, Montreal, QC, Canada; i Manipal McGill Centre for Infectious Diseases, Manipal Academy of Higher Education, Manipal, India

## Abstract

**Background:**

In India, men are more likely than women to have active tuberculosis but are less likely to be diagnosed and notified to national tuberculosis programmes. We used data from standardised patient visits to assess whether these gender differences occur because of provider practice.

**Methods:**

We sent standardised patients (people recruited from local populations and trained to portray a scripted medical condition to health-care providers) to present four tuberculosis case scenarios to private health-care providers in the cities of Mumbai and Patna. Sampling and weighting allowed for city representative interpretation. Because standardised patients were assigned to providers by a field team blinded to this study, we did balance and placebo regression tests to confirm standardised patients were assigned by gender as good as randomly. Then, by use of linear and logistic regression, we assessed correct case management, our primary outcome, and other dimensions of care by standardised patient gender.

**Findings:**

Between Nov 21, 2014, and Aug 21, 2015, 2602 clinical interactions at 1203 private facilities were completed by 24 standardised patients (16 men, eight women). We found standardised patients were assigned to providers as good as randomly. We found no differences in correct management by patient gender (odds ratio 1·05; 95% CI 0·76–1·45; p=0·77) and no differences across gender within any case scenario, setting, provider gender, or provider qualification.

**Interpretation:**

Systematic differences in quality of care are unlikely to be a cause of the observed under-representation of men in tuberculosis notifications in the private sector in urban India.

**Funding:**

Grand Challenges Canada, Bill & Melinda Gates Foundation, World Bank Knowledge for Change Program.

## Introduction

Multiple systematic reviews,^1^, ^2^, ^3^ including a review of 56 prevalence surveys from 24 countries, have found that men are more than twice as likely to have active tuberculosis but are considerably less likely than women to be diagnosed and notified to national tuberculosis programmes. In India, this represents a reversal of the usual pattern of disadvantage for women in use of health care.^4^, ^5^, ^6^, ^7^ Although India's national tuberculosis programme receives 1·9 notifications regarding men for every notification regarding women,^8^ tuberculosis population prevalence is even higher among men, suggesting that men access tuberculosis care at substantially lower rates than women.^1^, ^9^ Understanding the sources of this gender imbalance is crucial to identifying and treating the missing millions of patients with tuberculosis globally.^10^

After an individual develops active tuberculosis, they must traverse a process of care-seeking, diagnosis, linkage to treatment, treatment initiation, and notification to national tuberculosis programmes.^11^, ^12^ Men might face a disadvantage at any or all of these stages, and identifying the stage of the care cascade that contributes most to the relative undernotification of men could help to focus interventions on the most important gaps in care.

Of these stages, our study focuses on understanding gender imbalance in the diagnostic process. This stage is challenging to evaluate and represents a crucial point in care where changes in provider behaviour could improve case detection.^13^, ^14^ Additionally, gender differences in the quality of care delivered by health-care providers, whether biased against women or men, have important implications for health equity and social justice.

Incomplete data (eg, patient charts) on clinical processes in these settings combined with the complexity of the diagnostic process and differences in presentations between men and women makes it difficult to answer a conceptually simple question: when men and women with the same tuberculosis symptoms visit the same health-care providers, do they receive the same quality of care during initial clinical evaluations? In the absence of (complete) medical records,^15^ we use standardised patients to answer this question.

Standardised patients are people recruited from local populations and extensively trained to portray a predetermined and scripted medical condition to health-care providers. In India, standardised patients were first used to understand primary care provider practice in rural and urban India,^16^, ^17^ and their use has since expanded to a variety of settings and health conditions. Standardised patients are increasingly considered the gold standard for assessing the practice of health-care providers in low-income and middle-income countries.^18^, ^19^ For tuberculosis, our research team has validated the use of such patients in urban India, and subsequent research has extended the method to China, Kenya, and South Africa to assess quality of tuberculosis care.^20^, ^21^, ^22^, ^23^, ^24^

**Research in context**

**Evidence before this study**

In India, as in many other countries with a high tuberculosis burden, men substantially outnumber women among notified people in the national tuberculosis programme. However, men are usually underrepresented in these programmes relative to their share of the tuberculosis disease burden, meaning that men are disadvantaged at some point in the care seeking process relative to women. Whether health providers themselves contribute to these differences through delays in diagnosis (or misdiagnosis) of men or women is unknown. Reliance on administrative programme data and provider interviews is insufficient for discerning potential gender differentials, because of biases created from potentially different care-seeking patterns across gender. Therefore, how gender differences in notification rates reflect access to or provision of health services and differential quality of tuberculosis care for men and women is uncertain. The standardised, simulated patient method, which is considered the gold standard method to assess provider practice, allows us to rigorously address the quality of tuberculosis care by gender.

**Added value of this study**

The standardised patient method, by ensuring that the case presentations and provider selections of men and women are identical, is used here to determine whether a significantly different response occurs on the provider side when treating otherwise identical case presentations from men and women. Our study used a large, representative sample of private health-care providers in two urban Indian settings with standardised patients to compare the quality of tuberculosis care received by men and women. Because standardised patients were not assigned to providers with a random assignment function from a computer program, but instead by a field team blinded to this study, we did tests that confirmed that standardised patients were assigned by gender as good as randomly. We found that men and women do not receive different health-care experiences in terms of quality of care (ie, correct case management). Providers were as likely to correctly manage men as they were women, extending to several management decisions among all provider types.

**Implications of all the available evidence**

Systematic differences in quality of care are unlikely to be a cause of the observed under-representation of men in tuberculosis notifications in the private sector in urban India.

To understand gender differences in quality of tuberculosis care, we use the same data from our publication on quality of tuberculosis care in the private sector of two Indian cities.^23^ In that study, we documented wide variation in quality of care across providers that was poorly explained by location and education level, although it was highly responsive to case presentation.^23^ In this Article, we use the fact that our standardised patients included both men and women to examine systematic differences in care that could be attributed to gender.

## Methods

### Study design

We analysed clinical interactions done by standardised patients portraying four tuberculosis case scenarios at health facilities in the two Indian cities of Mumbai (population 12 million) and urban Patna (2 million).^25^ Health facilities were representatively selected in each city using random sampling described previously,^23^ stratified by qualification and pilot tuberculosis programme engagement status. The health facilities included providers across the range of qualifications available in urban India. On one end of this range were chest specialists and providers with Bachelor of Medicine, Bachelor of Surgery (MBBS) degrees. At the other were providers without an MBBS, including practitioners trained in ayurveda, yoga and naturopathy, unani, siddha, and homoeopathy, as well as registered medical practitioners and those without any formal medical training at all. In each city, stratified sampling was used to randomly oversample providers enrolled in pilot tuberculosis programmes in the private health sector.

### Procedures

Data collection for the study was done between Nov 21, 2014, and Aug 21, 2015, in both cities, as part of a larger study on tuberculosis care among private health-care providers in urban India.^23^ Details of the standardised patient method used in this study are discussed in our previous publications,^20^, ^21^, ^22^, ^23^ including our validation study of the standardised patient method for tuberculosis in India.^20^ The validation study^20^ showed that standardised patients were able to recall clinical encounters accurately, that detection of standardised patients as fake patients was low, and that the method posed no major risks to either providers or the standardised patients themselves.

Standardised patients portrayed four different standardised cases (table 1) developed with the support of a technical advisory group comprised of clinicians, public health experts, economists, anthropologists, and experts in both the Standards for TB Care in India (STCI) and the International Standards of TB Care.^26^, ^27^ Each standardised patient primarily portrayed only one of the four cases. Several standardised patients worked in both cities, and, in Patna, all standardised patients did some Case 1 interactions, even if this was not their primary presentation. All medicines prescribed or offered to the standardised patients were independently coded and classified. Further details regarding standardised patient recruitment, training, sampling, and data collection are provided in the appendix.

**Table 1:** Standardised patient case scenario descriptions

	**Case description**	**Presentation of patient**	**Expected correct case management**
Case 1	Classic case of presumed tuberculosis with 2–3 weeks of cough and fever	Presents with presumptive tuberculosis, for the first time, to a private health-care provider, saying “Doctor, I have cough that is not getting better and some fever too”	Recommendation for sputum testing, chest radiograph, or referral to a public DOTS centre or qualified provider
Case 2	Classic case of presumed tuberculosis in a patient who has had 2–3 weeks of cough and fever. The patient has taken a broad-spectrum antibiotic (amoxicillin) given by another health-care provider for 1 week with no improvement. He also carries an abnormal chest x-ray suggestive of tuberculosis	Presents after an initial, failed (empirical) treatment for symptoms with broad-spectrum antibiotics and a diagnostic chest x-ray, saying “I have cough and fever which is not getting better. I went to a doctor and took the medicines he gave me and have also had an x-ray done.” The chest x-ray and blister pack for the antibiotics are shown if the provider asks	Recommendation for sputum testing, chest radiograph, or referral to a public DOTS centre or qualified provider
Case 3	Chronic cough with a positive sputum smear report for tuberculosis from a public health facility	Presents with evidence of microbiologically confirmed tuberculosis, saying “I am having cough for nearly a month now and also have fever. I visited [the local government hospital] and they gave me some medicines and did a sputum test.” The sputum report is shown if the provider asks	Either referral to a public DOTS centre, a qualified provider or specialist, or (in the case of a qualified private provider) initiation of treatment with standard, four-drug, first-line anti-tuberculosis therapy (isoniazid, rifampicin, pyrazinamide, and ethambutol [the HRZE regimen])
Case 4	Chronic cough and, if asked, elaborates a history of previous, incomplete treatment for tuberculosis, which would raise the suspicion of multidrug-resistant tuberculosis	Presents as a previously treated patient with tuberculosis with recurrence of the disease (ie, suspicion of drug resistance), saying “Doctor, I am suffering from a bad cough. One year ago I had got treatment in [the local public hospital], and it had got better. But now I am having cough again”	Recommendation for any drug-susceptibility test (culture, line probe assay, or Xpert MTB/RIF) or referral to a public DOTS centre or qualified provider

DOTS=Directly observed treatment, short-course.

This study was granted clearance by the ethics committees at McGill University Health Centre in Montreal, Canada, and the Institute for Socio-Economic Research on Development and Democracy in New Delhi, India. All the standardised patients were hired as field staff and participated in training and refresher training to mitigate any potentially harmful events, such as injections, invasive tests, and consuming any medicines during encounters (appendix). As described in our previous publication that uses the same data,^23^ we sought a waiver of provider informed consent based on the research ethics provisions from the Government of Canada Panel on Research Ethics and a study commissioned by the United States Department of Health and Human Services to assess the ethics of simulated patient studies.^28^ Supported by our pilot study, which validated the use and ethical implementation of the standardised patient method for tuberculosis,^20^ both ethics committees approved the waiver, particularly for the following reasons: (1) combining informed consent with the congregation of providers during association meetings and the implementation of tuberculosis interventions during the study period posed threats to the scientific validity of the study objectives as well as to the risk of standardised patient detection, and (2) no more than minimal risk is associated with participation for the standardised patients or the providers, as reported in our validation study.^20^ All questionnaires and case scripts are available from the authors upon request.

### Outcomes

Table 2 shows the outcome measures that we used and how they were measured. Our primary outcome was correct case management (table 1), which is a predetermined, case-specific outcome benchmarked against the STCI and approved by the technical advisory group convened before standardised patient data collection.^26^ Secondary outcome measures included the following dimensions of process quality indicators: history questions asked, time spent with the patient, diagnosis provided, medicines given, whether the standardised patient was counselled on treatment, their assessment of whether the environment was private or distracting, whether they liked the doctor, whether they would go to the provider again, and whether the provider seemed knowledgeable and addressed their concerns seriously. We assessed the number and types of medications prescribed to the standardised patients for each of these cases. In addition to assessing the number of different medications prescribed or dispensed in an interaction, we also report the use of broad-spectrum antibiotics, anti-tuberculosis prescriptions, fluoroquinolone antibiotics, and steroids, which can have negative patient-specific or public health consequences. Treatment with fluoroquinolone antibiotics or steroids can mask primary tuberculosis symptoms, leading to a delay in accurate diagnosis.^29^, ^30^

**Table 2:** Standardised patient variable descriptions

	**Measurement method**
**Balance variable**	
MBBS provider	Recorded in provider data
Provider younger than 30 years of age	Assessed by standardised patient
Provider 30–50 years of age	Assessed by standardised patient
Provider older than 50 years of age	Assessed by standardised patient
Provider male	Observed by standardised patient
Patients waiting on arrival	Observed by standardised patient
Patients waiting on departure	Observed by standardised patient
Provider has clinic assistant	Observed by standardised patient
**Process indicator**	
Provider used cell phone	Observed by standardised patient
Other people in room during interaction	Observed by standardised patient
Television on during interaction	Observed by standardised patient
Essential checklist %	Calculated from standardised patient data
Time with provider (min)	Measured by standardised patient
Did the provider create a private environment?	Assessed by standardised patient
Did the provider explain about your illness?	Assessed by standardised patient
Did the provider explain your treatment plan?	Assessed by standardised patient
Did you like this doctor?	Assessed by standardised patient
Would you go to this doctor again?	Assessed by standardised patient
Did the provider seem knowledgeable about your illness?	Assessed by standardised patient
Did the provider address your worries seriously?	Assessed by standardised patient
How would you rate the provider? (1–10)	Assessed by standardised patient
**Quality outcome**	
Correct management	Calculated from standardised patient data
Referred case	Reported by standardised patient
Tuberculosis suspicion	Reported by standardised patient
Chest x-ray	Reported by standardised patient
Sputum acid-fast bacillus	Reported by standardised patient
Xpert MTB/RIF	Reported by standardised patient
Any medicine	Reported by standardised patient
Polypharmacy	Reported by standardised patient
Anti-tuberculosis treatment	Determined by analysis team
Fluoroquinolone	Determined by analysis team
Other antibiotic	Determined by analysis team
Steroids	Determined by analysis team

### Statistical analysis

A primary concern for the validity of the standardised patient method is that the individuals presenting the cases were not actually ill and, therefore, do not automatically present the correct clinical appearance, despite their training. To ensure that standardised patient presentations were convincing, such that detailed questioning and examination of the standardised patient did not lead providers to conclude that the standardised patients were healthy, we examined associations between case management and checklist completion for each standardised patient.

We did two sets of comparisons to establish that providers visited by women were, on average, identical to providers visited by men presenting the same standardised patient case scenario. In our study, women and men were recruited as standardised patients in all case scenarios in each city. However, fieldwork conditions precluded the explicit random assignment of standardised patients to providers, and the assignment of standardised patients to providers was done in the field by supervisors who were blinded to the fact that we would do a gender analysis. This made it highly unlikely that standardised patients who were men (or women) were assigned to providers with perceived higher or lower quality. Nevertheless, to verify that the assignment was as good as random, we used balance tests and placebo regressions as randomisation tests.

Specifically, we did a randomisation test to establish that we could obtain unbiased estimates of the effect of patient gender on quality of care outcomes by using placebo regressions based on standardised patient gender for each case. The rationale for this randomisation test is as follows: if standardised patient gender was correlated with provider quality within each case, then the providers who saw women present Case 1 should differ from those who saw men present Case 1 only within Case 1. By contrast, all other comparisons between those two groups of providers should be uncorrelated with that assignment. Therefore, for each case, we split the sample into the group of providers who saw any man present the case, and the providers who saw any woman present that case (these groups could overlap). Using these two groups, we ran six placebo regressions comparing how those two groups treated all other potential standardised patient cases (a total of 24 regressions). We report additional information regarding power calculations for such an audit study using standardised patients in the appendix.

We used ordinary least squares regression and logistic regression to assess differences in clinical care processes and case management across standardised patients by gender. In these specifications, we controlled for differences that arose from the study design. These included the location, the case scenario, and whether the provider had an MBBS qualification. We have shown previously that these differences affect the care that is provided and were components of our study design.^23^ We complemented ordinary least squares regressions with logistic regressions where appropriate, reporting odds ratios by standardised patient gender for dichotomous outcomes and illustrating appropriate CIs for these estimates. We clustered standard errors at the individual standardised patient level when calculating gender differences, and we used inverse-probability-weighted estimates based on our sampling strategy to arrive at city-representative interpretations of our outcome measures. Therefore, reported estimates correspond to the expected average quality of care outcomes and gender differences if providers were chosen at random by a patient from each city with each city contributing equal weight (appendix).^23^, ^31^ All data analyses were performed with Stata 15.

### Role of the funding source

The funders of the study had no role in study design, data collection, data analysis, data interpretation, or writing of the report. The corresponding author had full access to all the data in the study and had final responsibility for the decision to submit for publication.

## Results

2602 interactions were done by 24 unannounced standardised patients at 1203 different health-care facilities across two cities (table 3). 1900 (73%) interactions were done by men, who made up 16 of our 24 individual standardised patients (table 3). We describe the findings in three parts: (1) whether assignment of standardised patients in the field produced as good as random allocations of women and men for valid inference; (2) how the objective and subjective patient experience varied between women and men; and (3) how provider case management decisions and quality of care varied between women and men.

**Table 3:** Distribution of interactions and standardised patients by case, gender, and city

	**Case 1**		**Case 2**		**Case 3**		**Case 4**		**Total**
	Interactions	Standardised patients	Interactions	Standardised patients	Interactions	Standardised patients	Interactions	Standardised patients	
Patna (women)	191	5	69	1	73	2	79	1	412
Patna (men)	382	8	69	1	77	1	79	1	607
Mumbai (women)	77	2	127	1	33	1	53	1	290
Mumbai (men)	727	6	120	1	171	2	275	3	1293
Total	1377	..	385	..	354	..	486	..	2602

We found that increased clinical scrutiny was associated with higher propensity to treat the patient as though they had tuberculosis, which suggests that providers in general were convinced by the presentation of our standardised patients. A 100% completion rate for the essential history question checklist for each case was associated with a 4% (95% CI −7 to 16; p=0·443) change in the likelihood of correct treatment compared with no questions asked, a 14% (2 to 26; p=0·018) increase in the likelihood of giving any medication, an 18% (6 to 29; p=0·0032) increase in the likelihood of any verbal diagnosis, and a 16% (6 to 26; p=0·0011) increase in the likelihood of a verbal tuberculosis diagnosis. Although we cannot totally reject provider response to individually varying standardised patient characteristics on all outcomes, the measured height, weight, and age of the standardised patients jointly had no effect on correct management decisions (p=0·125) and their inclusion as controls does not systematically affect our main results, ruling out confounding due to gender-correlated physical attributes that might prompt clinical conclusions.

We found no differences in the provider's qualification, age, gender, caseload (measured by queue length at arrival and departure), or the presence of a clinic assistant (table 4).

**Table 4:** Balance test for interaction characteristics across standardised patient interactions

	**Women**		**Men**		**Adjusted difference (regression estimate)**	**95% CI**	**p value**
	N	Mean (SD)	N	Mean (SD)			
MBBS provider	702	0·56 (0·5)	1900	0·40 (0·49)	−0·05	−0·16 to 0·05	0·31
Provider younger than 30 years of age	702	0·04 (0·21)	1900	0·05 (0·21)	0·03	−0·02 to 0·07	0·24
Provider 30–50 years of age	702	0·68 (0·47)	1900	0·73 (0·44)	0·01	−0·07 to 0·09	0·85
Provider more than 50 years of age	702	0·27 (0·45)	1900	0·22 (0·41)	−0·03	−0·11 to 0·04	0·37
Provider male	702	0·89 (0·31)	1892	0·87 (0·34)	−0·02	−0·07 to 0·02	0·35
Patients waiting on arrival	702	2·60 (5·49)	1900	2·15 (4·53)	−0·17	−0·71 to 0·37	0·52
Patients waiting on departure	702	2·07 (4·19)	1900	1·88 (3·16)	−0·09	−0·42 to 0·24	0·58
Provider has clinic assistant	700	0·69 (0·46)	1898	0·61 (0·49)	−0·02	−0·07 to 0·04	0·55

This table reports the characteristics of providers in each of the 2602 presentations, comparing interactions completed by standardised patients who were men with those completed by women. It then reports linear differences and 95% CIs and p values for those differences. Reported differences are linear regression coefficients on the gender of the standardised patient, controlling for city and case scenario and standard errors are clustered at the individual standardised patient level.

Across the 19 comparisons for which we had sufficient statistical power for logistic regression (five pairs were not computable), one was significant at p<0·1, one at p<0·05, and one at p<0·01 (table 5). The two at p<0·1 and p<0·05 are expected by chance with 19 simultaneous comparisons and are, therefore, rejected as statistically insignificant by Bonferroni multiple-hypothesis, critical value adjustments. The one at p<0·01 occurred in a sample of 35 interactions, and our results are robust to their exclusion. This result is consistent with the assessment that women and men were as good as randomly assigned to providers during this study and reinforces our conclusion that women and men visited equivalent providers during the fieldwork. Consequently, observed differences in clinical interactions between women and men can be attributed to the gender of the standardised patient rather than variation in undetected provider differences.

**Table 5:** Randomisation test across providers who saw differently gendered case presentations

	**Saw a woman presenting**		**Saw a man presenting**		**Adjusted odds ratio (regression estimate)**	**95% CI**	**p value**
	Number of interactions	Correct management proportion	Number of interactions	Correct management proportion			
**Case 1**							
Case 2 (Women)	50	0·70	161	0·63	1·08	0·55–2·11	0·82
Case 2 (Men)	44	0·66	165	0·66	0·91	0·43–1·94	0·81
Case 3 (Women)	39	0·33	83	0·25	0·83	0·27–2·55	0·74
Case 3 (Men)	48	0·25	220	0·31	1·22	0·55–2·72	0·63
Case 4 (Women)	37	0·22	95	0·13	0·65	0·14–2·91	0·57
Case 4 (Men)	46	0·17	310	0·10	0·45	0·10–1·94	0·28
**Case 2**							
Case 1 (Women)	54	0·39	45	0·47	2·46	0·86–7·05	0·093
Case 1 (Men)	189	0·49	201	0·45	1·07	0·56–2·04	0·84
Case 3 (Women)	9	0·33	1	1·00	..	..	..
Case 3 (Men)	15	0·40	18	0·44	7·77	0·61–98·56	0·11
Case 4 (Women)	6	0·33	6	0·50	4·38	0·22–89·15	0·34
Case 4 (Men)	21	0·29	14	0·07	0·00	0·00–0·03	0·00048
**Case 3**							
Case 1 (Women)	41	0·39	52	0·42	2·13	0·54–8·38	0·28
Case 1 (Men)	105	0·45	253	0·43	1·28	0·61–2·66	0·52
Case 2 (Women)	9	0·44	13	0·69	10·72	1·03–111·07	0·047
Case 2 (Men)	1	1·00	17	0·76	..	..	..
Case 4 (Women)	3	0·33	11	0·45	..	..	..
Case 4 (Men)	13	0·23	29	0·31	1·58	0·46–5·37	0·46
**Case 4**							
Case 1 (Women)	37	0·43	43	0·40	0·62	0·15–2·60	0·52
Case 1 (Men)	101	0·48	336	0·35	0·66	0·31–1·39	0·28
Case 2 (Women)	6	0·83	18	0·72	2·64	0·14–48·29	0·51
Case 2 (Men)	6	1·00	14	0·50	..	..	..
Case 3 (Women)	3	0·00	10	0·40	..	..	..
Case 3 (Men)	12	0·58	28	0·39	0·49	0·13–1·90	0·30

For each case scenario, this table shows a test of balance across the providers who saw a man present that case and the providers who saw a woman present that case. For each other gender-case presentation, it assesses whether any significant difference exists between those two groups of providers. The table presents the N, mean correct management proportion, odds ratio, 95% CI, and p value for differences in correct management between those two groups. Reported odds ratios are logistic regression coefficients on the gender of the standardised patient, controlling for city, case scenario, and provider qualification, and standard errors are clustered at the health care facility level.

We compared process indicators by standardised patient gender as well as the standardised patients' own subjective experiences of the interactions (table 6). We observed one significant difference between the reported experiences of women and men: providers spent significantly less time with men (8·24 min *vs* 5·57 min; 95% CI −3·69 to −1·59; p<0·0001). On all other observed dimensions we found no differences in interactions by standardised patient gender.

**Table 6:** Differences in interaction process indicators by patient gender

	**Women**		**Men**		**Difference**	**95% CI**	**p value**
	N	Mean (SD)	N	Mean (SD)			
Provider used cell phone	700	0·13 (0·34)	1900	0·10 (0·30)	−0·02	−0·07 to 0·03	0·42
Other people in room during interaction	700	0·17 (0·37)	1900	0·14 (0·35)	−0·05	−0·12 to 0·02	0·18
TV on during interaction	700	0·03 (0·17)	1900	0·03 (0·17)	0·00	−0·02 to 0·03	0·78
Essential checklist %	702	0·50 (0·25)	1900	0·50 (0·25)	−0·01	−0·12 to 0·11	0·93
Time with provider (min)	698	8·24 (6·19)	1894	5·57 (3·60)	−2·64	−3·69 to −1·59	<0·0001
Did the provider create a private environment?	700	0·70 (0·46)	1900	0·74 (0·44)	0·03	−0·07 to 0·14	0·48
Did the provider explain about your illness?	700	0·10 (0·30)	1900	0·07 (0·26)	−0·03	−0·2 to 0·13	0·66
Did the provider explain your treatment plan?	700	0·28 (0·45)	1900	0·21 (0·41)	−0·08	−0·24 to 0·08	0·30
Did you like this doctor?	700	0·85 (0·35)	1900	0·82 (0·39)	−0·04	−0·13 to 0·06	0·45
Would you go to this doctor again?	700	0·83 (0·38)	1899	0·78 (0·42)	−0·04	−0·16 to 0·07	0·46
Did the provider seem knowledgeable about your illness?	700	0·59 (0·49)	1900	0·43 (0·49)	−0·10	−0·2 to −0·01	0·042
Did the provider address your worries seriously?	700	0·61 (0·49)	1900	0·42 (0·49)	−0·23	−0·36 to −0·11	0·00087
How would you rate the provider? (1–10)	700	6·85 (2·04)	1900	6·05 (2·23)	−0·71	−1·4 to −0·01	0·046

This table reports estimated differences by gender across individual interactions as observed or assessed by the standardised patient during or after the interaction. It then reports linear differences and 95% CIs and p values for those differences. Linear differences are estimated controlling for city and case scenario and standard errors are clustered at the individual standardised patient level. All variables are binary, except time with provider (expressed in min), the provider rating (1–10), and essential checklist (%).

Comparisons of the standardised patients' subjective assessment of quality, however, show that men were less likely to agree that the provider seemed knowledgeable about the illness or that the provider addressed their worries seriously (table 6). Men also rated the providers lower than did women on a subjective scale (table 6). Under the assumption that the men did not have different initial perceptions or attitudes towards the providers, the subjective assessment mirrors the shorter interactions with men.

Additionally, differences occurred in the types of history questions asked of women and men (appendix). Men were more likely to be asked about smoking (with estimates ranging from 12 percentage point difference to 24 percentage point difference, depending on case) and drinking (3–18% percentage point difference, depending on case) habits, whereas women were more likely to be asked about children (16–37 percentage point difference; appendix). Questions related to the tuberculosis diagnosis, however, did not vary systematically across the cases. Only small differences (directionally inconsistent across cases) occurred in the frequencies of essential questions like the duration of cough or whether the cough produces sputum (appendix). For physical examinations, there were small differences, with women more likely to have had their blood pressure taken (appendix).

Overall proportions of correct management were 40% for women and 36% for men (table 7). We detected no differences in this measure of STCI-compliant management or in any other key quality dimensions of care, such as medication use and laboratory testing, between women and men. All estimated differences in correct case management were statistically insignificant, of small absolute magnitude, and in varying directions by case (table 7). Estimates ranged from 3% less correct case management for men relative to women in Case 4, to 5% more in Case 1, highlighting the absence of any systematic difference in correct case management by gender (table 7). These differences remained insignificant in all subsamples—MBBS providers, non-MBBS providers, whether the provider was a man or a woman, or within either study city.

**Table 7:** Differences in quality of care by patient gender

		**Women**		**Men**		**Difference**	**95% CI**	**p value**
		N	Mean	N	Mean			
Correct management		702	0·40 (0·49)	1900	0·36 (0·48)	0·01	−0·02 to 0·05	0·47
	Case 1	268	0·40 (0·49)	1109	0·39 (0·49)	0·05	−0·06 to 0·16	0·32
	Case 2	196	0·64 (0·48)	189	0·68 (0·47)	0·04	−0·04 to 0·12	0·15
	Case 3	106	0·28 (0·45)	248	0·31 (0·46)	−0·01	−0·06 to 0·04	0·63
	Case 4	132	0·15 (0·36)	354	0·10 (0·30)	−0·03	−0·07 to 0·01	0·14
	MBBS provider	391	0·47 (0·50)	763	0·57 (0·50)	0·04	−0·04 to 0·11	0·34
	Non-MBBS provider	311	0·33 (0·47)	1137	0·21 (0·41)	−0·02	−0·13 to 0·08	0·69
	Male provider	628	0·39 (0·49)	1639	0·36 (0·48)	0·02	−0·03 to 0·06	0·52
	Female provider	74	0·49 (0·50)	253	0·33 (0·47)	−0·05	−0·18 to 0·08	0·41
	Patna	412	0·33 (0·47)	607	0·39 (0·49)	0·01	−0·03 to 0·05	0·67
	Mumbai	290	0·50 (0·50)	1293	0·34 (0·47)	0·02	−0·04 to 0·08	0·56
Referred case		702	0·09 (0·28)	1900	0·07 (0·26)	0·01	−0·04 to 0·06	0·64
Tuberculosis suspicion		702	0·41 (0·49)	1900	0·37 (0·48)	0·04	−0·07 to 0·14	0·47
Chest x-ray		702	0·42 (0·49)	1900	0·40 (0·49)	0·04	−0·06 to 0·13	0·45
Sputum acid-fast bacillus		702	0·21 (0·41)	1900	0·13 (0·33)	−0·03	−0·09 to 0·03	0·26
Xpert MTB/RIF		702	0·05 (0·22)	1900	0·04 (0·19)	−0·01	−0·03 to 0·01	0·18
Any medicine		702	0·83 (0·38)	1900	0·87 (0·33)	−0·02	−0·07 to 0·03	0·38
Polypharmacy		702	3·29 (2·03)	1900	3·76 (2·04)	−0·22	−0·48 to 0·04	0·089
Anti-tuberculosis treatment		702	0·07 (0·25)	1900	0·04 (0·19)	−0·01	−0·06 to 0·04	0·68
Fluoroquinolone		702	0·17 (0·37)	1900	0·11 (0·31)	−0·01	−0·05 to 0·04	0·71
Other antibiotic		702	0·45 (0·50)	1900	0·48 (0·50)	−0·06	−0·14 to 0·02	0·16
Steroids		702	0·12 (0·32)	1900	0·17 (0·38)	0·01	−0·02 to 0·04	0·35

This table reports estimated differences by gender across management decisions in individual interactions as determined by the analysis team. It then reports linear differences and 95% CIs and p values for those differences. Linear differences are estimated controlling for city, case scenario, and provider qualification, and standard errors are clustered at the individual standardised patient level. The eight subsets reported under correct management report the results of the correct management difference regression among the individual case scenarios and among subsets of providers by qualification and provider gender. All variables are binary, except polypharmacy (the whole number of medications given).

We also found no differences in any of the individual treatment behaviours composing the correct management index, including the decision to refer the case, whether the provider mentioned a suspicion of tuberculosis to the patient, or the choice among various types of tuberculosis testing. Similarly, we find no qualitatively large or statistically significant (at the 5% level) differences in the use of unnecessary medications. The overall absence of any large difference in case management outcomes is not an artifact of sample size or estimation methods—the null effects are precisely estimated with narrow confidence intervals and are robust to hypothesis testing using logistic regression (figure).

**Figure:**
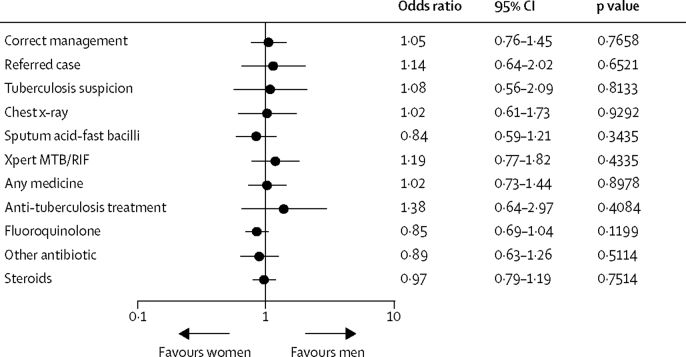
Differences in quality of care by standardised patient gender This figure illustrates estimated differences by gender across management decisions in individual interactions as determined by the analysis team. Odds ratios are estimated controlling for city, case scenario, and provider qualification, and standard errors are clustered at the individual standardised patient level. All variables are binary.

## Discussion

Our study assessed differences by gender in Indian health providers' management of tuberculosis patients with identical clinical profiles, using the gold-standard standardised patient method. We sought to understand whether gender-related differences in quality of care could be contributing to the relative under-diagnosis of men (and, therefore, their under-representation in notifications) with active tuberculosis in the general population, as has been found in previously published literature.^1^, ^9^ We demonstrate a general absence of differences in provider behaviour between men and women presenting symptoms of tuberculosis in a high-burden setting with high levels of background gender inequality and large gender differences in both tuberculosis prevalence and notification rates.

Three characteristics of the study provide a unique opportunity to assess gender differences in tuberculosis diagnosis and treatment among Indian health-care providers. First, the study is at-scale across two cities with 2602 patient-provider interactions across 1203 health care providers. Second, the study is representative: in each city, we used a comprehensive list of all private health-care providers to randomly sample providers stratified by qualification and reweighted outcomes for representative estimates. Third, the assignment of standardised patient gender among providers was as good as random, although it was not explicitly randomised. Women and men were hired as standardised patients in the study, and the gender of the standardised patient who presented each case to each provider was determined by the field team supervisors. These supervisors were blinded to the gender analysis in the study described here and to provider characteristics other than their name. Although the randomisation was not done explicitly by the researchers, we tested that the assignment of standardised patients to providers in the field resulted in an allocation that was equivalent to explicit randomised assignment and statistically uncorrelated with provider characteristics.

These characteristics allowed us to estimate unbiased differences between provider treatment of women and men presenting identical tuberculosis case scenarios across a wide range of patient experience and provider treatment outcomes and to attribute any differences to the gender of the standardised patient. The standardised patient design is not confounded by gendered variation in case presentation across real patients, controls for selective choice of providers by patient gender, eliminates the social desirability response biases inherent in studies based on interviews with patients or providers, and is not susceptible to Hawthorne effects on provider behaviour.^9^, ^32^, ^33^

Variations by gender in history taking and consultation process seem to be primarily social and quantitatively small. We did not observe systematic differences in provider practice on any quality measures, for any case presentation, or for any level of provider qualification. Additionally, we find no evidence that providers of either gender behave differently when matched with patients of the same gender as themselves. Poor quality of care during the initial clinical evaluation for individuals with suspected tuberculosis, which we have discussed previously, affects women and men equally.^20^, ^21^, ^23^, ^34^, ^35^ However, men appear to have received care that involves less provider time and less detailed explanation, and reported significantly worse perceptions of the providers' knowledge, seriousness, and overall satisfaction.

We cannot measure in our study whether any of these differences would lead men or women with tuberculosis symptoms to have different experiences of patient satisfaction or stigma on average, potentially shaping future care-seeking behaviour and engagement in tuberculosis care, although this is a distinct possibility. Systematic reviews and multisite studies show that in many settings, including India, men are less likely to complete tuberculosis therapy, more likely to die during treatment, and more likely to experience disease recurrence after completing treatment.^3^, ^36^, ^37^ As such, understanding whether gender-related differences in the time spent by providers, in the explanations given to patients, and in patient satisfaction remain consistent during subsequent stages of care, and whether these differences contribute to men's poorer tuberculosis outcomes, is an important area for future research.

The strength of the study design and scale of implementation in the two large cities located in different regions of India, lead us to believe that the lack of systematic gender differences in the management of tuberculosis patients in urban India, including appropriate management, referral rates, choice of diagnostic, or use of unnecessary medication is a key and robust finding. Our null findings covered a broad range of process indicators and quality outcomes and have narrow confidence intervals around zero. Our strong balance and randomisation tests suggest that these estimated null results are unlikely to be confounded by observed or unobserved factors. Although we observed gender differences in history taking and examination, as well as differences in time spent with the patient and subjectively reported satisfaction, these differences appeared to have little consequence for case management.

Nevertheless, our study had several limitations. First, although the study was a population-weighted assessment of average behaviours for these provider types and cities, it was not necessarily statistically representative of the provider mix that patients face if women and men choose to visit different types of providers on average and might not replicate in other settings. Second, in this study, observed practice only reflected what health-care providers did when they came across a completely unknown or new patient seeking medical care in their first visit to the health care provider. Third, this study only covered private practitioners in two urban areas in India.

Additionally, inherent limitations exist in our approach of using standardised patients to assess gender differences. Although each standardised patient visited over 100 facilities on average, the decision to hire 24 individual standardised patients was based on fieldwork logistics and supervision constraints, which has implications for the power and bias in this study (discussed further in the appendix). Specifically, if gender and other disadvantages intersect (eg, care might be demonstrably worse for low-caste men), our study is externally valid only to the extent that these other disadvantages were also represented in our standardised patient selection.

We have compared the characteristics of our standardised patients with profiles of tuberculosis patients who visit private clinics in urban India using NFHS-4, which is a nationally representative sample of 601 509 households.^38^ Our standardised patients had similar age and education profiles, but no standardised patients were from the lowest wealth quintiles (17·6%), less than primary education (38·7%) and are children or elderly patients (34·9%). Therefore, our study only relates to the experience of 50–60% of patients who are in the middle and above wealth quantile, have secondary or higher education, and are between 18 and 59 years of age.

Standardised patient assignment was not randomised, so the credibility of the differences being due to gender is based on our evidence that the standardised patients were assigned as good as randomly across providers. Additionally, the standardised patient method is designed to provide objective estimates of actual provider behaviour; however, it provides little insight into why we observe gender-related differences in time spent with men. Detailed qualitative and ethnographic studies involving interviews with, and observation of, patients and providers might provide further insights into the social context that shapes these gender disparities in tuberculosis care.^39^, ^40^

Despite these limitations, the major findings from this study have implications for public health. Our main results suggest that concerns about health-care providers being responsible for gender differences in diagnostic delays are unlikely to be well-founded, though less time and explanation given by providers to men on average could adversely affect outcomes for men in subsequent stages of the care cascade. Our findings should not be taken to imply that neither men nor women experience disease-related stigma or unique challenges in seeking or accessing tuberculosis care, but they do show that they do not face a systematic gender-related difference in care quality from health providers during the initial diagnostic evaluation for tuberculosis.^9^, ^41^, ^42^

As we discussed previously in multiple contexts regarding the supply-side of health care, the main cause for concern is the low overall level of correct management for all patients, and its lack of strong correlation with provider characteristics like qualifications.^21^, ^22^, ^23^ For women and men, the average health-care provider did not ask the essential history questions that would lead to a tuberculosis diagnosis, did not mention tuberculosis suspicion to the patient (although this might be generally acceptable due to concerns of stress and stigma or caution ahead of receiving more convincing evidence), and did not order appropriate microbiological tests to diagnose tuberculosis as per the STCI and international recommendations. Instead, more than 80% of interactions resulted in medicine prescriptions, half of which contained unnecessary antibiotics that do not have a role in tuberculosis care and have negative public health consequences. Prescriptions of fluoroquinolone antibiotics and steroids are particularly worrisome, as they can mask tuberculosis symptoms, leading to delays in diagnosis.^30^

Given the lack of gender-related differences in quality of care delivered by providers, how can the relative under-notification of men be explained? As described previously, an individual with tuberculosis must traverse three stages to start treatment: care-seeking, diagnostic evaluation, and linkage to treatment. The absence of gender differences in diagnostic evaluation suggests potential barriers for men at the other two stages. Men might be less likely than women to seek care in the first place for their tuberculosis symptoms or to link to tuberculosis treatment after diagnosis in the Indian context. Although systematic reviews have summarised the literature in India on care-seeking, delays in reaching care, and linkage to treatment, they and many of the included studies did not specifically evaluate gender differences for each.^11^, ^12^ Additionally, few of the studies included in those reviews evaluated patients seeking tuberculosis care in the private sector.

As such, research is needed to better understand gender differences across these other stages of the tuberculosis care cascade and to inform policy and programmes. The standardised patient method might be extended to assess gender differences in quality of tuberculosis care in other countries and in India's public sector. In addition to evaluating the diagnostic workup for tuberculosis, the standardised patient method might also have utility for understanding gender differences in quality of care during tuberculosis treatment initiation. The standardised patient method is less useful for understanding gender differences during later stages of the care cascade that involve longitudinal follow-up, given the risk that standardised patients might be detected by providers. For these later stages, quality of care can potentially be measured using cohort studies to understand gender differences in patient outcomes and provider behaviour patterns, especially with regard to linkage to treatment, treatment completion, and recurrence-free survival. Additionally, future research to understand the issue of gender differentials in tuberculosis case notifications might focus on infection risks, the availability and accessibility of high-quality providers, and the decision making of symptomatic individuals.

## Data sharing

Individual de-identified interaction data, including data dictionaries, will be available. All variables needed to recreate the results reported in this article will be included, as will the code required to reproduce these results. Data will be available indefinitely upon publication to anyone who wishes to access the data for any purpose. The data and code can be accessed at https://github.com/qutubproject/lancetgh2019.
