# Supplementary material for: Use of standardised patients to assess gender differences in quality of tuberculosis care in urban India: a two-city, cross-sectional study
Source: Lancet Glob Health. 2019 Mar 27;7(5):e633–43. doi: 10.1016/S2214-109X(19)30031-2 (PMC6465957; doi:10.1016/S2214-109X(19)30031-2)
Supplement: Supplementary appendix [file mmc1.pdf]

THE LANCET
Global Health

Supplementary appendix

This appendix formed part of the original submission and has been peer reviewed. We post it as supplied by the authors.

Supplement to: Daniels B, Kwan A, Satyanarayana S, et al. Use of standardised patients to assess gender differences in quality of tuberculosis care in urban India: a two-city, cross-sectional study. *Lancet Glob Health* 2019; published online March 27. http://dx.doi.org/10.1016/S2214-109X(19)30031-2.

Supplementary Appendices to:
Gender Differences in Quality of Tuberculosis Care in Urban India:
A two-city, cross-sectional study

Benjamin Daniels, MSc†[1], Ada Kwan, MHS†[1,2], Srinath Satyanarayana, MD, PhD†[3], Ramnath Subbaraman, MD[4], Ranendra K. Das, PhD[5], Veena Das, PhD[6], Jishnu Das, PhD‡[1,7], and Madhukar Pai, MD, PhD‡[8,9]

[1]Development Research Group, The World Bank, Washington DC, USA
[2]University of California at Berkeley, Berkeley, USA
[3]Center for Operational Research, International Union Against TB and Lung Diseases, Paris, France
[4]Department of Public Health and Community Medicine, Tufts University School of Medicine, Boston, USA
[5]Institute for Socio-Economic Research on Development and Democracy, Delhi, India
[6]Department of Anthropology, Johns Hopkins University, Baltimore, USA
[7]Center for Policy Research, New Delhi, India
[8]McGill International TB Centre, McGill University, Montreal, Canada
[9]Manipal McGill Centre for Infectious Diseases, Manipal Academy of Higher Education, Manipal, India

† Contributed equally
‡ Contributed equally

Corresponding author:
Prof Madhukar Pai, MD, PhD
Canada Research Chair in Epidemiology Global Health
McGill University
Dept of Epidemiology Biostatistics
1020 Pine Ave West
Montreal, QC H3A 1A2, Canada
Email: madhukar.pai@mcgill.ca

A1 Fieldwork Details

Note: The following section is an edited version of the Supplemental Appendices from Kwan et al. (2018) and Das et al. (2015), tailored for this study.

A1a Description of SP Case Scenarios

Four tuberculosis (TB) case scenarios were developed to document the level and variation in quality of care for TB among sampled providers. For each case, both the clinical case presentation and social contexts were developed and agreed upon by a technical advisory group, which included clinicians, economists, anthropologists, experts in international and national TB guidelines, and other stakeholders. The four cases were:

1. **Case 1 (Naïve TB Suspect)** – A classic case of presumptive TB with 2-3 weeks of cough and fever. The SP presents to the providers and begins the interaction with the opening statement: "Doctor, I have a cough that is not getting better and some fever too."

2. **Case 2 (TB Suspect with Abnormal Chest X-Ray)** – A classic case of presumptive TB who has had 2-3 weeks of cough and fever, a history of completed chest X-ray and 1 week of broad-spectrum antibiotic treatment ordered by another provider, with no improvement. The SP carries a digital chest X-ray dated within the last 10 days with evidence of abnormalities, and the blister pack of amoxicillin with him/her. The SP begins the interaction by saying: "Doctor, I have had cough and fever. It is not getting better, even though I went to a doctor and took medicines also."

3. **Case 3 (TB Case)** – A chronic cough with a positive sputum smear report for TB from a public health facility. The SP carries the sputum microscopy test report and displays it prominently on his/her lap, mentioning that he/she has had his/her sputum tested. The SP begins the interaction by saying: "I am having a cough for almost a month now and also have a fever. I visited the Government hospital, and they gave me some medicines and did sputum tests."

4. **Case 4 (MDR Suspect)** – A classic case of presumptive TB with 2-3 weeks cough and fever, and, if asked, a history of previous incomplete TB treatment, which would raise the suspicion of multi-drug-resistant TB. The SP begins the interaction by saying: "Doctor, I am suffering from a bad cough. One year ago, I got treatment in the Government hospital, and it had got better, but now have a cough again. I went back to the same hospital, and they did a sputum test." In 50 additional interactions in Mumbai, the SP is randomly assigned

to carry a TB-positive sputum test report as in Case 3 and displays it prominently on his or her lap.

A1b SP Recruitment, Script Development, and Training

SP Recruitment

For the two-city study presented here, 24 individuals (8 women and 16 men) were recruited and hired as SPs, including both new recruits and individuals. The SP cohort in each city was comprised of a different set of individuals with 13 of the 24 individuals hired for the study in Patna and 17 of the 24 individuals hired as SPs for the study in Mumbai. Some SPs had prior experience as they had participated in our validation study in Delhi (Das et al. 2015) and/or other SP studies assessing quality for other health conditions aside from TB.

During the recruitment process, all potential SPs underwent a health screening questionnaire and checkup, and all SPs in the final cohorts were seemingly healthy, which meant they had no apparent health conditions that could confound the case presentation and interaction with providers. The SPs, although recruited specifically to fit each case scenario and corresponding narrative, differed in age, gender, height, and weight. The average age of all the SPs was 30. The youngest was 21; the oldest was 39. The 16 men weighed 50 to 74 kilograms and were 160 to 184 centimeters tall. The 8 women weighed 46 to 72 kilograms and were 147 to 160 centimeters tall.

For Patna data collection (21 November 2014 to 28 February 2015), the SP cohort consisted of 13 individuals (5 women), who conducted interactions with MBBS or higher and non-MBBS providers. For these interactions 6 SPs (2 women) were Case 1, 2 SPs (1 woman) were Case 2, 3 SPs (2 women) were Case 3, and 2 SPs (1 woman) were Case 4. All SPs in Patna conducted some Case 1 interactions, regardless of their primary assignment. All SPs were originally from the State of Bihar, of which Patna is the capital.

In Mumbai, 17 individuals (5 women) conducted fieldwork between 2 April 2015 to 21 August 2015. For these interactions, 8 SPs (2 women) were Case 1, 2 SPs (1 women) were Case 2, 3 SPs (1 woman) were Case 3, and 4 SPs (1 woman) were Case 4. SPs were originally from the States of Bihar (5), Madhya Pradesh (1), and Maharashtra (11). Primary languages spoken by the SPs included: Angika (1), Bangali (1), Bhojpuri (1), Hindi (4), Magahi (2), and Marathi (8).

SP Script Development [1]

Each SP case scenario described in S1a was coupled with a script. Each script is a narrative that describes the social and family contexts of the patient. The scripts were developed under the

[1] The following text is an edited version of the Supplemental Appendices from Kwan et al. (2018) and Das et al. (2015), tailored for this study.

guidance of an anthropologist (VD) with active supervisor and SP participation. Together, the case scenarios and scripts were piloted in our validation study in Delhi as presented in Das et al. (2015). They were again refined based on field and data management lessons from Delhi and again during and after training in Patna and Mumbai for the study presented in this paper.

The two most important considerations for script development that were also tightly linked to SP training were: First, the clinical symptoms and case history had to reflect the social and cultural milieu of which the SP was assumed to be a member, and second, the presentation of symptoms and answers to history questions had to be consistent with biomedical facts about the disease.

On the former, SPs brought a lot of socially appropriate understanding of the local vocabularies through which symptoms were to be presented and also about typical life histories that would correspond to the age, gender, caste, religion and class of the character that the SP was portraying. As a simple but crucial example, people among the strata the SPs were drawn from do not often use thermometers to measure temperature but report fever on the basis of the sensation of heat and rapid pulse. The inputs by SPs in script development were crucial from this perspective.

The latter issue was to train SPs to present symptoms and answer questions pertaining to case history that were medically correct. For example, all opening statements and questions pertaining to the type of cough and its duration were standardized. A critical part of the training was to help SPs distinguish between questions to which answers could be improvised but had to be appropriate to the social role of the SP and answers that had to be given using local idioms but in a standardized format without any alterations.

The dual aim of presenting the disease in a manner that was not misleading and avoiding detection were largely successful because the reasoning behind both objectives was carefully and repeatedly explained to the SPs and because of their active involvement in the script development and hands-on training. SP case scripts are available from the authors upon request.

SP Training [2]

To portray the four SP cases, the 24 otherwise healthy individuals recruited as SPs were trained in each city to finalize the case presentation given their knowledge of context, internalize the scripts and cases, be able to debrief with a supervisor within 1-2 hours of the interaction, and present in clinical settings in a way that would avoid any potentially harmful risks and detection. Thus, SP training was designed with four specific aims:

1. To ensure the SPs correctly present the cases in a standardized way;

[2]The following text is an edited version of the Supplemental Appendices from Kwan et al. (2018) and Das et al. (2015), tailored for this study.

2. To ensure the SPs accurately recall the interaction that occurred with health care providers;
3. To ensure SPs avoid both detection or any suspicion that the interaction was not genuine; and
4. To ensure SPs are prepared to avoid potentially harmful risks that can occur to them.

The first two aims were achieved through extensive classroom training in case presentation and recall. Classroom training was complemented with mock interviews and followed by live supervised dry runs in the field at clinics not sampled for the study. Our pilot study in Delhi described in Das et al. (2015) also included the use of tape recorders in a selected subset of interactions, which we used to verify that the results reported on the structured questionnaires were accurate reflections of the clinical interactions.

For the third aim, SPs were carefully instructed to avoid detection by the following methods. First, our recruitment strategy ensured that SPs came from low-income areas or slums from the same cities in which the project was located, so they could easily pass for genuine and local patients, but the areas from which they came were located far from the field sites, so they would not be personally known in the areas they conducted interactions. Second, supervisors for SP fieldwork traveled into the field for 'scoping exercises' before any of the SP interactions were conducted. These scoping exercises helped supervisors to familiarize themselves with landmarks, clinic locations and addresses, general setting, operating hours, length of potential wait time or queues, need for clinic appointments, and other potential issues that could pose challenging to the SP interaction. Based on scoping, the team ensured that SPs were set up to conduct as-successful-as-can-be interactions. Third, during the training, time was organized such that SPs could internalize the characters for each case and the details of their mock stories through which the character was made alive to them. In mock interviews during training, supervisors added unscripted questions with regard to the patient's family or neighborhood details, which SPs were able to answer spontaneously because they were of the actual social background that was being approximated in the characters they were portraying. Finally, dry runs were conducted in which the supervisor was present in the shop on the pretense of buying something, such as toothpaste or an over-the-counter cough syrup, and thus could watch the interaction and use additional training time to improve the SPs' presentations of the cases.

For the fourth aim, SPs participated in active discussions on risk mitigation strategies. Together with the supervisors, the SPs brainstormed what they could do to mitigate risks or avoid situations that could be abnormal. They were then extensively trained on these and additional risk mitigation strategies. (Communication on these matters were prioritized and extended into the data collection period. Throughout the data collection period, which was weathered with fog that reduced visibility

to several meters, cold temperatures, scorching heat at times above 43°C/110°F, high humidity, annual monsoons, flooded roads, medicine bans, and elections, supervisor and SP meetings would occur near weekly. These meetings provided additional opportunities to discuss potential issues as a team and how to mitigate them as they were encountered in the field.)

In Patna, SPs were trained initially to present various cases to chemists, and after the completion of chemist interactions, an additional 5-day training was conducted for all the SPs to train them for Cases 1-4 and the associated risk mitigation techniques. Dry runs were conducted in both cities for interactions with providers. The dry runs also provided an opportunity to test the chest X-rays (CXRs) and corresponding reports carried by Case 2 and for the sputum reports carried by Case 3. Different sets of abnormal CXRs for both women and men were procured by ISERDD from a contact of a member of the QuTUB team (SS). The digital CXRs were shipped from Hyderabad throughout fieldwork approximately every ten days. Sputum reports were researched and produced by the field team.

In Mumbai, all of these individuals took part in a comprehensive and rigorous 10-day training, which included 3 days of dry runs. The entire standard training was done in Hindi, since even Marathi-speaking individuals were expected to encounter doctors speaking in Hindi, and after classroom training, Marathi-speaking SPs went over scripts and exit questionnaires in Marathi, followed by dry runs in Marathi. Dry runs were conducted outside of the wards selected for the sample. There were several full meetings for SPs and supervisors, and refresher trainings were conducted every time a new schedule was provided to the field team, approximately every three weeks.

A1c Rationale for Approved Waiver of Provider Informed Consent

Ethics guidelines on health service audit studies state that SPs should be used in cases where the person being sent the SP is providing a service to other people and where other options have been carefully studied but cannot answer the research questions required. In addition, there should be minimal risk to the participants. Based on 10 years of research, we have demonstrated the difficulties of obtaining quality of care data without using SPs (Alderman et al., 2014). In previous SP studies, we have requested and received waivers of informed consent from ethics committees at Johns Hopkins University, Harvard University, and Duke University. Another SP study conducted at the Universidad Peruana Cayetano Herediahas has also received waiver of informed consent. These waivers have been granted under the provisions for waiver or alteration of the informed consent requirements under the United States Department of Health and Human Services regulations 45 CFR 46.116(d) [3].

[3] Office for Human Research Protections (OHRP). Accessed at: http://www.hhs.gov/ohrp/policy/consentckls.html.

Although the ideal study design would include informed consent, we sought a waiver because (a) we were sending multiple SPs to the same healthcare provider, and (b) we would be carrying out these assessments as part of a quality of care surveillance for a TB program (PPIA) being implemented by NGOs in Mumbai and Patna. In this case, the consequences of potential detection were very high, as the entire 3-year study could have been jeopardized. Further, as part of the PPIA program, providers who were part of the PPIA network were to attend trainings and workshops together. This added a risk to the SP study - for example, if providers in Mumbai and Patna were consented for an SP study, the PPIA networked providers could discuss the identities and personal characteristics of the SPs. The combination of informed consent and congregation of providers at frequent intervention trainings (at times several are scheduled in one week) threatened the validity of our study as reported responses would not reflect the actual quality of care we were aiming to measure, while increasing the risk of SP detection.

We therefore worked closely with Institutional Review Board (IRB) requirements on informed consent, which is handled as per the provisions of the Government of Canada Panel on Research Ethics in the 2nd edition of the Tri-Council Policy Statement of Ethical Conduct for Research Involving Humans' Article 3.7 entitled "Alteration of Consent in Minimal Risk Research" [4]. Prior to the current study, we conducted a pilot with informed consent to validate the SP method for tuberculosis in urban India (published as Das et al. (2015) in Lancet Infectious Diseases). The results of the pilot validation study confirmed the decision to seek a waiver of informed consent in the current study. Corresponding to the requirements of Article 3.7, we documented in our pilot study that the SP approach in urban India was no more than minimal risk of participation to the SPs or providers. This was based on the following considerations:

1. Opinion data from providers in the pilot demonstrated that participation in the study did not adversely affect their practice in any way.

2. No monetary loss was incurred by the providers as the SPs, like real patients, paid the full consultation fee.

3. No added inconvenience was placed on real patients as the SPs were trained to immediately step aside if there were an emergency that demanded the doctor's attention.

4. None of the identities of the providers or their clinics were compromised since we maintained strict anonymity in the information collected and never disclosed the identity of health care providers who participated in the study.

5. From our observations, average consultation times in our pilot were between 3 and 7 minutes,

[4] Government of Canada Panel on Research Ethics. 2012. TCPS 2 – Chapter 3, Accessed at: http://www.pre.ethics.gc.ca/eng/policy-politique/initiatives/tcps2-eptc2/chapter3-chapitre3/

so that would only inconvenience other patients by that time.

Based on these considerations, we requested a waiver of informed consent from the IRB at ISERDD and the Research Ethics Board at McGill University. The requests for a waiver were reviewed and approved by both ethics committees, contingent upon the provision of a letter of full disclosure to be sent to debrief any provider who received an SP at the end of the study. The letter will offer health care providers a chance to further discuss any aspect of the findings or methodology and register any concerns; however, no individual data on any clinic or provider will be disclosed. Because our larger quality of care surveillance study has been extended to December 2019, this letter will be circulated thereafter.

A1d Provider Sampling

During the primary data collection period for this study in Mumbai and Patna, urban TB programs funded by the Bill and Melinda Gates Foundation (BMGF) and implemented by Private Provider Interface Agencies (PPIAs) in each city - World Health Partners (WHP) in Patna and PATH in Mumbai - were mapping, recruiting, and enrolling private sector providers into provider networks in both cities. At the time of sampling for our study in these two cities, we decided to stratify our provider sample by PPIA program enrollment as detailed in Appendix Figure A1. From lane-by-lane mapping exercises conducted by the PPIA, which resulted in a universe list of private sector providers in Mumbai and Patna, we obtained lists of enrolled and not-enrolled providers in the PPIA program and merged them to produce a complete sampling universe stratified by PPIA enrollment status. We then restricted these lists based on eligibility criteria for the SP study: providers eligible for the study were those who were known to see adult outpatients with respiratory symptoms in the private health sector. The description of the program serves to support sampling weights (Table 1) applied to achieve the urban area estimates for Mumbai and Patna but stratified findings based on PPIA program enrollment are not presented in this paper.

Patna

The WHP field team conducted a street-by-street mapping exercise between January and July 2014 in urban Patna. The exercise involving mapping all health facilities and providers, as well as pharmacists and laboratories in the private health sector. Areas covered included all wards in Danapur, Phulwari Sharif and Patna blocks in Patna District. The resulting lists, along with PPIA program engagement lists and program activity data, became the raw files for the SP study sampling frames in Patna. Based on the provider qualifications captured during the WHP mapping exercise, providers were categorized by qualification (MBBS and higher, non-MBBS) for the purpose of the SP study. De-duplication of mapping entries was achievable based on the fol-

lowing mapping data fields: provider name, facility name, provider telephone, block, ward, and address. The non-MBBS provider list was relatively easy to clean; however, the MBBS or higher provider list was not in useful shape, which was confirmed in the field. To verify the accuracy of addresses, ISERDD and several authors of this study conducted several scoping exercises before and throughout data collection.

On December 5, 2014, BMGF and WHP initiated a second mapping of formal providers to replace the cleaned formal provider universe list, for the purpose of SP study sampling and program targeting activities. The second mapping of formal providers was particularly advantageous for the SP study for four reasons. It allowed us to (1) see what proportion of non-engaged providers were actually relevant for the PPIA program, (2) ensure that details relevant for the SP study were included in the mapping for the MBBS or higher provider assessment, (3) ensure that good information was captured for relocating the providers for when the PPIA field team would begin sensitization, engagement, and training activities and for when the SPs were sent to them, and (4) do as much as possible to avoid any field issues, such as rare operating hours, clinics open one day a week, and sending SPs to providers not eligible for the study, such as child specialists or orthopedists.

We, the quality of tuberculosis care (QuTUB) team, worked with WHP's M&E officer and had the support of ACCESS Health International (AHI). The mapping activity was planned for ten medical officers and supervised by a field supervisor who would report to the rural and urban M&E officers. The mapping was completed at the beginning of February 2015. The mapped MBBS and higher provider universe was delivered to the QuTUB team in two parts, since the SP field team was waiting for an updated schedule of interactions. Because the updated mapping universe was delivered in two parts, the sampling for non-engaged MBBS and higher providers was broken into two strata: MBBS and higher providers mapped between December 5 and January 9, and MBBS and higher providers mapped from January 10 to beginning of February. There were no significant differences between these two groups (results available upon request).

At the end of both mappings, the cleaned universe in Patna consisted of 1,841 formal providers and 1,338 informal providers (see **Appendix Figure A1**). Patna SP interactions occurred between 21 November 2014 and 28 February 2015. SP interactions were done first for informal (non-MBBS) providers, followed by formal (MBBS or higher) providers.

Mumbai

Between January and June 2014, PATH contracted out street-by-street mapping activities in Mumbai of private health facilities, originally planned for 12 high TB burden wards and 3 high-slum population wards, which account for 86% of the Mumbai slum population and 70% of the Mumbai

population. The mapping activity was done by two community-based organizations (Alert India and Maharashtra Janavikas Kendra (MJK)). Mapping was initiated in G-North (high TB burden), M-East (Chembu slums and presence of Chest Physicians), P-North, and S wards. By June 2014, the exercise resulted in a provider universe of 8897 locations in 18 wards, which were 12 fully mapped wards, partial mapping of H-East, P-South, and R-South wards, and minimal mapping in the remaining wards. Of the 8897, 2804 were chemists, 3591 were AYUSH practitioners (BAMS, BUMS, BHMS, DAMS, DHMS, LCEH), 671 less than fully qualified allopathic providers, 1290 MBBS, 463 MDs, and 78 chest physicians. Similar to Patna, a provider universe list produced by the IMS Health company was reviewed, but a decision within the PATH team and separately within the QuTUB team was made not to use or append this list.

Since the network established by PATH aimed to move with the providers regardless of where and in how many facilities they practiced, the final QuTUB sampling frames were the result of thorough cleaning, scoping, and verification exercises in collaboration with the PATH team. Before fieldwork began, an individual (R) hired by ISERDD together with a representative from AHI (NC), both from Mumbai, heavily scoped the field to check for addresses and to gather additional information that would be useful before the ISERDD field team moved to the city. The information that was gathered included, but was not limited to: identifying potential SP recruits, looking at the transportation systems, reporting the setup of clinics and health facilities, collecting GPS locations of providers, and correcting provider and facility names while capturing consulting fees, first points of contact in a facility, and outpatient department (OPD) timings.

Four wards out of the 15 original focus wards of the PPIA program were selected for the majority of Mumbai SP surveillance to minimize geographical spread of the SP study. This was because increasing the geographic scope of the city would increase logistical difficulties as presented by Mumbai's transit system and scale. In mid-January 2015, a meeting was conducted between the PATH and QuTUB teams to agree on the four study wards. After assessing upcoming PPIA program efforts, access to transportation, mixture of unregistered and registered slums, and available data on the wards (e.g., total ward population, slum population, and proportion of slum population figures as per the 2011 census), F-North, K-East, L, and P-North were selected. Relative to other mapped wards, these four wards have a higher slum population (with the exception of F-North), and they also have more providers and more networked providers. Together, these wards were a good representation of areas for PPIA Mumbai efforts. As noted in sections below, these wards were used for constructing the AYUSH (see **Appendix Figure A1**) and non-PPIA facility ("hub") samples.

In addition to PPIA hubs, there were non-networked private hospitals and single provider private clinics that met the PPIA hub criteria (i.e., in-house or nearby digital X-ray, pharmacy,

and chest physician) and could provide a reasonable comparative 'apples-to-apples' estimate of the quality of major facilities not enrolled in the program. The list of non-PPIA hubs was created with the assistance of PATH, who verified whether each one would be a comparable candidate for enrollment into the program. The list consisted of health facilities that met the criteria for the PPIA network and located in the four study wards: F-North, K-East, L, and P-North wards. An initial list of eligible but non-PPIA 'hubs' was created by PATH in January 2015, and this list was revisited and updated in April 2015. The process of updating the list at the end of April 2015 involved PATH team members and FOs going through the list and removing any facilities that had been networked since July 2015, when SP interactions began for the eligible, yet non-PPIA hubs.

The FOs also went through the full universe list again to make sure the PPIA networking criteria still applied, and any duplicates were removed (at least 12 sets of duplicates or triplicates). The final list was frozen on April 28, 2015, and the QuTUB team flagged facilities that were ineligible for the SP study (i.e., children hospitals) before establishing the sampling frame for non-PPIA hubs. The final list contained 78 eligible, yet non-PPIA hubs, and 11 were excluded from actual SP visits, since they were children or orthopedic hospitals and the SP cases did not reflect pediatric TB or extrapulmonary TB (see **Appendix Figure A1**).

Furthermore, we ascertained how PPIA providers (MBBS and higher) practice at PPIA hubs and non-PPIA locations. To expand on this, it was common for individual PPIA providers practicing at PPIA hubs to also have other private practices, which we deem as 'non-PPIA locations' or 'non-networked locations'. These are locations where the providers were confirmed to not be networked regardless of being networked in the PPIA at a different location. To confirm the PPIA status of these private practices, an initial mapping with verification activities for these 'non-networked locations' occurred between April and July 2015. For initial mapping, two PATH team members in collaboration with the QuTUB team procured a list of practices for 150 PPIA providers, who were MBBS, MDs, and MD/Chest Physicians and who were all networked at known PPIA hubs (see **Appendix Figure A1**). Of the 150, 136 met the inclusion criteria (i.e., providers who see adult pulmonary patients) for the SP study.

In terms of the process, FOs conducted the non-networked location mapping in two phases. The first phase, which was circulated to the QuTUB team on July 3rd, contained 153 locations for 62 of the 136 eligible providers and was collected from FOs in R South, N, G North, M East, M West, and F North wards (providers were interviewed at their networked location linked to a ward and a corresponding FO). This resulted in a list of non-networked locations for 62 providers where: 18 providers had 1 location; 21 providers had 2 locations; 10 had 3; 6 had 4; 4 had 5; 2 had 6; and 1 had 7. PATH team members reviewed these locations to determine their networked statuses, and AHI and ISERDD teams conducted scoping for eligibility into the SP study (e.g., government

facilities were ineligible for the SP study). The second phase contained details for the remaining 74 providers. For the verification activities, ISERDD and AHI (NC) did on-ground scoping to double-check for any government facilities, ensure there were no duplicates, confirm outpatient hours of the providers, and check whether providers had on-call duties at any of the locations. At the time of verification, ISERDD had already completed all PPIA and non-PPIA hub walk-ins, and favorably, the team was also able to map whether or not any of these non-networked locations had already been visited as a non-PPIA hub. If so, facility IDs matching with those non-PPIA hubs were mapped back to the sample and analytically used also for 'non-networked' interactions.

A1e Assignment of SP Cases to Providers

Patna

Two different samples were randomly generated for non-MBBS providers:

1. Uniform-probability random sample of non-PPIA, non-MBBS providers (with reserves)

2. Uniform-probability random sample of PPIA, non-MBBS providers (with reserves)

Drawn from the full provider universe of all wards in Danapur, Phulwari Sharif, and Patna blocks of urban Patna (source: WHP mapping data), the random samples for the PPIA and non-PPIA samples for non-MBBS providers were restricted to the following geographical space: 40/40 wards of Danapur block, 28/28 wards of Phulwari Sharif block, and 34/73 wards. These wards were purposively selected areas of Patna block and decided in collaboration with WHP to cover areas which could have higher TB burdens - Kankarbagh, Rajendra Nagar, Patna City, and Patliputra Housing Colony neighborhoods. PPIA engagement status used for sampling was frozen on September 25, 2015.

MBBS and higher providers were also selected with stratified uniform-probability random sampling. Engagement status and program activity was frozen on September 25, 2014. The two samples were:

1. Uniform-probability random sample of non-PPIA, MBBS and higher providers

2. Uniform-probability random sample of PPIA-engaged, MBBS and higher providers

Similar to the non-MBBS geographical sampling, the random samples for MBBS providers were drawn from the same geographical areas as the random sample for non-MBBS providers. One field challenge surmounted in December 2014 for the non-PPIA, MBBS sample, which resulted in halting the fieldwork in December 2014. A quarter of the interactions attempted resulted in specialists irrelevant and ineligible for the study, and others had closed shop and moved since the WHP mapping exercise. Since WHP initiated a new mapping activity for MBBS and higher providers

in the same month, as described in the mapping section, we took advantage of this to resample this group, as well as to conduct a pre-screening selection for an impact evaluation. Since the re-mapping and SP fieldwork coincided, we re-sampled the non-PPIA, MBBS and higher group in two waves as the mapping data arrived: strata 1 and 2. Replacing the non-PPIA, MBBS and higher sample, Stratum 1 contained 150 providers and Stratum 2 contained 100 providers. A total of 500 total assigned interactions were attempted, of which 442 were completed. There were no significant differences between the two strata (available upon request).

Additionally, on January 7, 2015, 120 MBBS and non-MBBS providers who had already received an SP portraying Case 1 were scheduled another Case 1 portrayed by a different SP actor. A four-week grace period at minimum between the first visit and the beginning of the revisit interaction period was designed to minimize detection. The objective of this was to see how consistent providers were with patients of identical case presentation. Case 1 revisits were scheduled after the first non-engaged, MBBS provider schedule was halted and before the non-engaged MBBS sample could be replaced from WHP remapping. This meant that a subset of non-MBBS and MBBS providers received a second Case 1, regardless of PPIA or non-PPIA engagement status and regardless of sampling strategy. For the Case 1 revisits, ISERDD used the full cohort of 12 SPs while finishing up any remaining interactions left over from the previous informal and engaged formal samples.

Mumbai

Non-MBBS sampling (see **Appendix Figure A1**) was restricted to AYUSH practitioners and to four study wards as agreed with PATH. Engagement statuses of AYUSH were frozen on January 24, 2015. There were three samples that resulted:

1. Uniform-probability random sample of non-PPIA AYUSH in K-East and L wards
2. Uniform-probability random sample of non-PPIA AYUSH in F-North and P-North wards
3. Census of PPIA AYUSH in all four wards

Schedules were given to ISERDD in two groups: AYUSH in K-East and L wards in one group, and AYUSH in F-North and P-North wards in a second group. For both groups, reserves were provided for the non-PPIA practitioners based on ward. All AYUSH interactions were randomly assigned to be conducted either in the morning or evening hours.

Next, general walk-ins without appointments to hubs were conducted by having the SP enter the health facility and go to the doctor suggested by the receptionist or intake nurse, without regard for PPIA status of the provider, at both PPIA-registered facilities and non-PPIA facilities. There were two samples:

1. Non-PPIA hub walk-ins at purposively selected locations in the four wards

2. PPIA hubs walk-ins (census of all PPIA hubs) across 15 wards

All hub walk-ins were conducted during specific times that were given to the field team. For PPIA hubs specifically, walk-ins were conducted during hours any networked doctor were scheduled to practice, unless an appointment was scheduled by the receptionist or intake nurse. If, during the consultation, the SP was told to go to another doctor at that moment, the SP was instructed to do so and any aspect of the interaction was also recorded on the same form. On May 1, 2015, before sending SPs to the field, ISERDD supervisors met with the PATH team to go over all the PPIA hubs, their locations, the facility layout, and the networked providers.

Walk-ins were first conducted among 100 PPIA hubs. Only Case 1 interactions were conducted. Once PPIA hub walk-ins were completed, walk-ins were done at non-PPIA hubs. Since we had to still confirm whether these facilities would accept a presumptive TB walk-in patient, all non-PPIA hubs were sent an SP portraying Case 1. Only after Case 1 interactions were successfully completed, we randomly assigned half to receive Case 2 and then randomly assigned another half to receive either Case 3 or Case 4. None of the SPs trained as Case 4 carried a sputum report to the non-PPIA hubs. To avoid any risk of the same SP running into the same provider across locations, the following field protocols were put in place: (1) SPs were to remain cognizant and maintain a list of providers they had visited, and (2) supervisors were to review a provider directory when assigning SPs for interactions. At least two PPIA providers were seen by SP1 during the non-PPIA walk-in, while 54 of 93 PPIA facility walk-ins resulted in the SP seeing a PPIA provider.

We then targeted additional PPIA providers at facilities where they practiced as part of the PPIA (see **Appendix Figure A1**). Updated lists for providers, mostly MDs and MD Chest Physicians, networked by the PPIA were procured in January 2015 and then again in April 2015. A total of 136 providers at PPIA hubs were eligible for the SP study (ineligible providers, such as pediatricians and orthopedist specialists, were excluded) across 100 health facilities, resulting in 98 successful interactions. When scheduling the providers and locations that would receive SPs, our top priority was to maximize the total number of interactions we could conduct while reducing risk of detection. In order to do this, we had to work around three issues: (1) providers who were networked at multiple locations, (2) PPIA hubs that had more than 3 networked providers, and (3) providers selected who had already been seen during the walk-in visits. Selecting the eligible providers was done manually and checked several times.

Among the 136 providers, we were aware of 19 who were networked at multiple locations (13 providers at 2 facilities, 2 at 3, 4 at 4 = 48). To maximize the total number of interactions, we then selected these 19 providers at the facility that had the fewest number of other practicing doctors. Among the PPIA hubs, there were six hubs considered as large, defined as having more than 3 networked providers (2 hubs with 5 providers, 1 with 7, 1 with 9, 1 with 10, 1 with 11 = 57). To

avoid detection, we decided that no PPIA hub would receive SPs for more than three providers. In order to select the three providers at the large hubs, we attributed the eligible networked doctors to the number of TB cases they had reported to the PPIA program and ranked them from highest to lowest. We then removed all the providers located at multiple locations since we had already selected another one of their networked locations. Three providers were then selected by taking the highest notifying, the lowest notifying, and a random provider who had not been selected yet and who could be either notifying or not to the program.

All PPIA providers at PPIA hubs received SPs portraying Case 1 (however, if SPs as Case 1 had already seen the networked doctor during a walk-in, we did not assign another Case 1 to this doctor, and the assumption was the walk-in observation for Case 1 could be used for the provider targeted interaction for Case 1). A random half received Case 2. Another random half received Case 3 with a sputum report in addition to Case 4 without a sputum report, with the other half was assigned to not receive Case 3 and to receive Case 4 with a sputum report. To minimize detection risk, the field team conducted interactions at the six large facilities last.

Finally, we specifically targeted PPIA providers at their other practice locations where they were not considered part of the PPIA (non-networked locations or "NNLs"). From the verified list of non-networked locations, NNLs that were government facilities and outside Mumbai city were excluded for sampling. Some of the interactions, though not deliberately scheduled for ISERDD, took place during the walk-ins at non-PPIA locations when providers who also practiced within the network were encountered by chance. Scheduling for ISERDD was done in 4 waves as the mapping was done in parallel to fieldwork, and verification process was also done with SP1 case.

PPIA providers at NNLs were scheduled to receive Case 1 before any other cases. This was done to serve as a final verification for location eligibility. Case 1 was randomly assigned to one NNL for the providers who SPs visited in the previous sample at a networked location. If the provider was known to have multiple NNLs, we gave preference to the location where he or she was not on-call or working at an actual large PPIA hub before random selection, since large PPIA hubs had already received at least 3 provider-specific SP1s. All on-call locations and other NNLs for each provider was made available as a reserve list to the field team. All PPIA providers at their NNLs were assigned Case 1. The half that did not receive Case 2 at the networked location were assigned to receive them at the NNL. The half that received Case 4 with a sputum report were assigned to receive Case 4 without a sputum report.

A1f Medication Coding

After SP interactions were completed with sampled facilities in both cities, a list of all labeled medicines prescribed or offered to the SPs was independently coded and classified by two doctors

with expertise in TB (SS) and infectious diseases (RS). Medicine received as unlabeled and in loose pills form, such as those received by some AYUSH practitioners in Mumbai, were placed into small plastic bags by the SP field team with one bag for each pill type (defined as same color and shape) and marked as unlabeled on the SP exit questionnaire. We employed two pharmacists to independently identify these pills. On the basis of their assessments we determined whether the medicines given included at least one antibiotic or steroid.

References

1. Alderman H, Das J, Rao V. Conducting ethical economic research: complications from the field. In: DeMartino G, McCloskey D, editors. The Ox- ford Handbook of Professional Economic Ethics. Published online; 2014. Accessed at: http://www.oxfordhandbooks.com/view/10.1093/oxfordhb/9780199766635.001.0001/oxfordhb-9780199766635-e-018.

2. Das J, Kwan A, Daniels B, Satyanarayana S, Subbaraman R, Bergkvist S, et al. Use of standardised patients to assess quality of tuberculosis care: a pilot, cross-sectional study. The Lancet Infectious Diseases. 2015;15(11):1305–13. doi: 10.1016/S1473-3099(15)00077-8. PubMed PMID: 26268690.

3. Kwan A, Daniels B, Saria V, Satyanarayana S, Subbaraman R, McDowell A, Bergkvist S, Das RK, Das V, Das J, and Pai M. Variations in the quality of tuberculosis care in urban india: A cross-sectional, standardized patient study in two cities. PLOS Medicine, Forthcoming.

4. Satyanarayana S, Kwan A, Daniels B, Subbaraman R, McDowell A, Bergkvist S, et al. Use of standardised patients to assess antibiotic dispensing for tuberculosis by pharmacies in urban India: a cross-sectional study. The Lancet Infectious Diseases. 2016;16(11):1261-8.

A2 Statistical Methods

A2a Weighting

Based on our sampling strategy in **Appendix A1**, our city-level estimates of the quality of care in Mumbai and Patna extrapolate from the sampling frame to the full population of private health care providers in each city. To calculate averages and differences within and across cities, we utilize inverse probability weights to satisfy the following:

1. Each city-case combination (Patna Case 1, Mumbai Case 2, etc) has a total sum of weights equal to one. Therefore each case is equally weighted within each city, and the two cities have equal total weights.

2. Within each city-case combination, the sum of weights for (A) MBBS-qualified and above and (B) non-MBBS-qualified providers is exactly equal to each group's prevalence in the city as a whole.

3. Within each city-case-qualification group, the relative total weights for (A) PPIA and (B) non-PPIA providers are exactly proportional to each group's prevalence in that city and qualification stratum.

By satisfying these conditions, the weight on each interaction is calculated such that our estimates take the values that they would if we had sampled exactly at random from the city as a whole, assuming that our sample is representative of that provider mix. There are 32 weighting groups: one for each city, case, qualification, and PPIA status ($2 * 4 * 2 * 2$). Under the assumption that the providers we sampled from our sampling frames are representative of similar providers throughout the city, the resulting estimates are representative of the choice of a random provider within the city for each case presentation. When we report statistics of the form "X of Y interactions (N%)", Y is the whole number of interactions observed, X is the whole number of interactions in which the outcome occurred, and N is the population-level estimate calculated using the weights detailed above.

A3 Supplementary Results

A3a Results for all cases, disaggregated by city and SP gender

Table A1 reports fully disaggregated outcomes by city and gender, for each of the case management behaviors with gender differences estimated in **Figure 1**. 95% confidence intevals are reported in brackets below the means.

A3b Differences between provider history-taking by SP gender

Table A2 details item-by item differences in physical examination and history taking for men and women SPs. The first panel, for all SPs, assesses differences in physical examination. The remaining four panels, starting with Case 1 and proceeding through Case 4, enumerates the history questions checklist for that case. Then the table reports N, mean item completion, and estimated linear differences between the completion levels for women's and men's presentations of that case. Stars indicate conventional significance levels, ie * = $p<0.1$; ** = $p<0.05$; and *** = $p<0.001$.

A3c External Validity and Power Calculations for SP Audit Studies

Although this was a large SP study with over 1,200 providers assessed for differential treatment of men and women presenting with symptoms of TB, one limitation to the study is the fact that only 24 individual standardized patients participated. This is a large number for an SP study, but because this study focuses on a characteristic which is *fixed for each SP* – their gender – we take the time to discuss here the external validity and power of the study design. To date, we have identified no complete analytical treatment of this study design; here, we begin by addressing these issues conceptually and provide simulation results that cover our experiment only.

In terms of external validity, the primary concern is whether the estimates of differential outcomes for women and men we obtain here are applicable to the real patient population. Specifically, are the differences between provider treatment of men and women SPs likely to be the same as those in the general population? This cannot be empirically tested, because we cannot sample SPs at random from the general population. SPs are very selectively hired and extensively screened and trained for the ability to perform as an SP. However, as SPs are regarded as the gold standard for determining precise levels of quality of care by providers, differences between SP measurements are also valid comparisons. We also emphasize that SPs are drawn as much as possible from the ordinary working population of the areas in which they then seek care in our study.

In terms of power, the noise in the gender parameter estimate is critical because we report *no* significant differences, and we report that we also in general reject the possibility of large differences between women and men *because our standard errors are small.* If we significantly underestimated

the amount of noise in our estimates, our results would in effect have no evidentiary value, because they would not reject the possibility of large differences in provider treatment between patient genders. A recent survey of audit studies (**Table A3**) shows many with a large number of locations, with the very largest similar in size to this study – but only 2-4 testers in general. (Vuolo, Uggen, & Lageson 2016) We were unable to locate a study that systematically addressed the relationship between the number of auditors or SPs and the precision of the estimates.

Therefore, we conduct a precision simulation in which we assess a large number of "simulated studies". We assume a *population of potential SPs* and a *population of providers*. Then, each simulated study draws some (large) number of providers and some (small) number of SPs, matches them randomly, and for each "simulated interaction", creates an outcome where the gender of the SP has some effect on the providers' behavior. We analyze the variance of several estimators for the gender difference, choosing combinations of total number of SPs (4-100), split 50-50 by gender, and the number of providers (200-1600) and determining the needs for an accurate estimate of the population differences. Each size of study is randomly evaluated 100 times and averaged.

Over a large number of these simulated studies, we describe how accurate the measurement of the (true) gender difference is and how different estimators perform with different numbers of SPs and providers. By construction, all estimators in this simulation are unbiased on average for the true gender differential in the patient population. Our concern is that with a small number of SPs, chance idiosyncratic variations in the sample of SPs who are hired for each study – one SP who looks sick somehow, one SP who is unusually attractive – will cause us to measure this parameter less precisely than standard regression theory predicts. For each batch of 100 simulated studies for each size combination, we record (a) the true sampling variation in the point estimate of that parameter; (b) the average analytically calculated standard errors from an unadjusted regression; and (c) the average analytically calculated standard errors from a regression with cluster-robust standard errors at the individual SP level.

We compare these for performance in **Figure A2**. The upper-left panel of this figure illustrates the true way in which the point estimate of a single fixed parameter (in this case, SP gender) is subject to sampling error as the sample sizes for health care facilities and individual SPs change. At these sample sizes, the true estimation noise around the parameter of interest is decreased relatively quickly by increasing the number of individual SPs. The lower-left panel illustrates how ordinary, unadjusted regression estimates instead incorrectly report that the sampling noise is quickly reduced by increasing the number of facilities. The upper-right panel illustrates how the typical cluster-robust standard error adjustment produces accurate values for the noise in the parameter estimate, once the number of SPs is at or above the range of 15-20.

Finally, the lower-right panel illustrates how these three measures compare as the number of

individual SPs increases. The true amount of combined SP *and* provider sampling noise (the size of the standard errors around the true gender parameter) is underestimated by the unadjusted regression at all possible numbers of individual SPs, due to the fact that gender is fixed at the SP level. By contrast, the clustered estimator converges to the true values when approximately 15-20 individual SPs are included in the study, although it also underestimates the noise when the number of SPs is smaller than that. In this sense these initial results behave very similarly to a cluster-randomized control trial with the SPs serving as treatment clusters.

If this is the correct general model, then the extent to which the correct cluster-robust standard errors for audit study estimates in SP studies differ from the ordinary regression confidence intervals *(and therefore the relative importance of having more individual SPs versus having more facilities in the study)* depends on the degree to which outcomes are fixed for the individual SP – the intra-cluster correlation (ICC). The simulation values are reported for a simple simulation in which individual SP intra-cluster correlations are 0.33 (ie, equally contributing to outcome variance with an identically sized facility effect and a pure noise parameter). In our sample, we observe intra-cluster correlations of about 0.12, and correspondingly the unadjusted estimates of confidence intervals for our regressions are substantially closer to the correct (clustered) estimates that we report. We ascertain that this is so by re-running the regressions without clustering and obtaining similar standard errors on the same point estimates (not reported).

In general, as the SP method is adapted to audit studies such as this one, we hope that general methods for calculating correct standard errors are developed so that studies can be appropriately designed with the correct number and type of SPs and provider populations in mind.

References

1. Vuolo M, Uggen C, Lageson S. Statistical power in experimental audit studies: Cautions and calculations for matched tests with nominal outcomes. Sociological Methods & Research. 2016 May;45(2):260-303. Accessed at: http://users.soc.umn.edu/~uggen/Vuolo_Uggen_Lageson_SMR_2015.pdf

Figure A1: Sampling flow diagram by primary strata

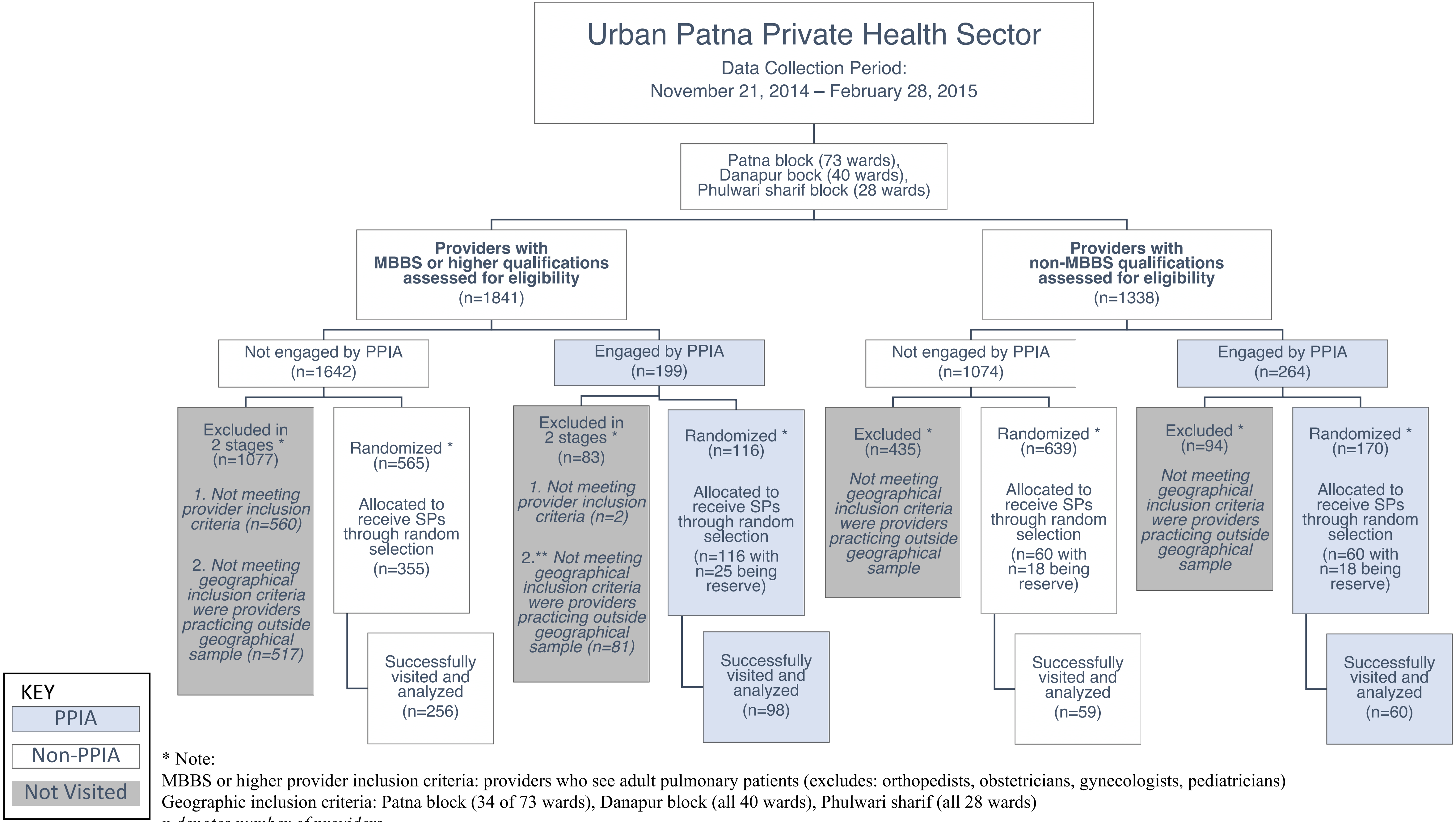

* Note:
MBBS or higher provider inclusion criteria: providers who see adult pulmonary patients (excludes: orthopedists, obstetricians, gynecologists, pediatricians)
Geographic inclusion criteria: Patna block (34 of 73 wards), Danapur block (all 40 wards), Phulwari sharif (all 28 wards)
n denotes number of providers

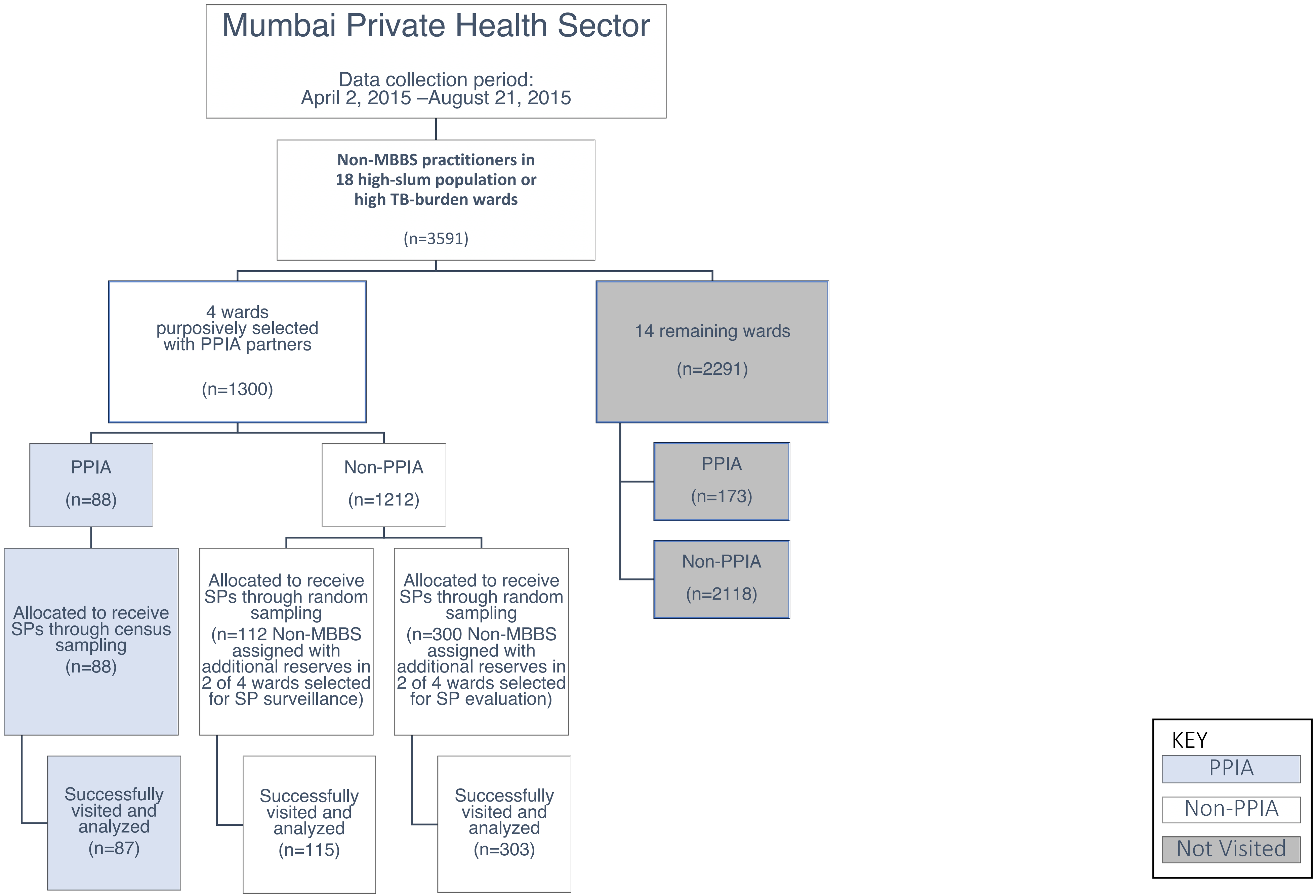
Mumbai Private Health Sector
Data collection period:
April 2, 2015 –August 21, 2015
Non-MBBS practitioners in 18 high-slum population or high TB-burden wards
(n=3591)
4 wards purposively selected with PPIA partners
(n=1300)
14 remaining wards
(n=2291)
PPIA
(n=88)
Non-PPIA
(n=1212)
PPIA
(n=173)
Non-PPIA
(n=2118)
Allocated to receive SPs through census sampling
(n=88)
Allocated to receive SPs through random sampling
(n=112 Non-MBBS assigned with additional reserves in 2 of 4 wards selected for SP surveillance)
Allocated to receive SPs through random sampling
(n=300 Non-MBBS assigned with additional reserves in 2 of 4 wards selected for SP evaluation)
Successfully visited and analyzed
(n=87)
Successfully visited and analyzed
(n=115)
Successfully visited and analyzed
(n=303)
KEY
PPIA
Non-PPIA
Not Visited

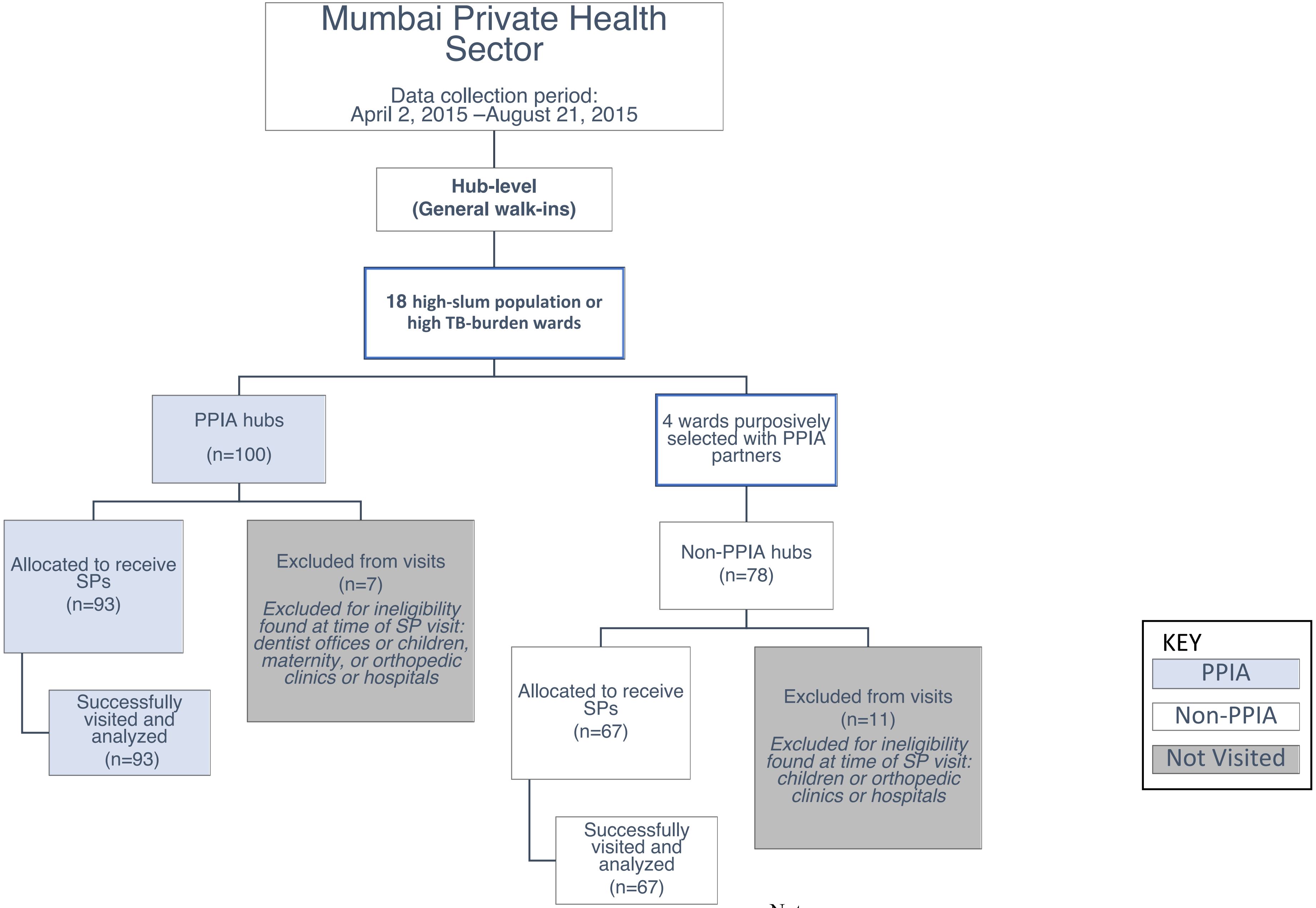

Note:
n denotes number of private health facilities (overlaps with PPIA provider interactions)

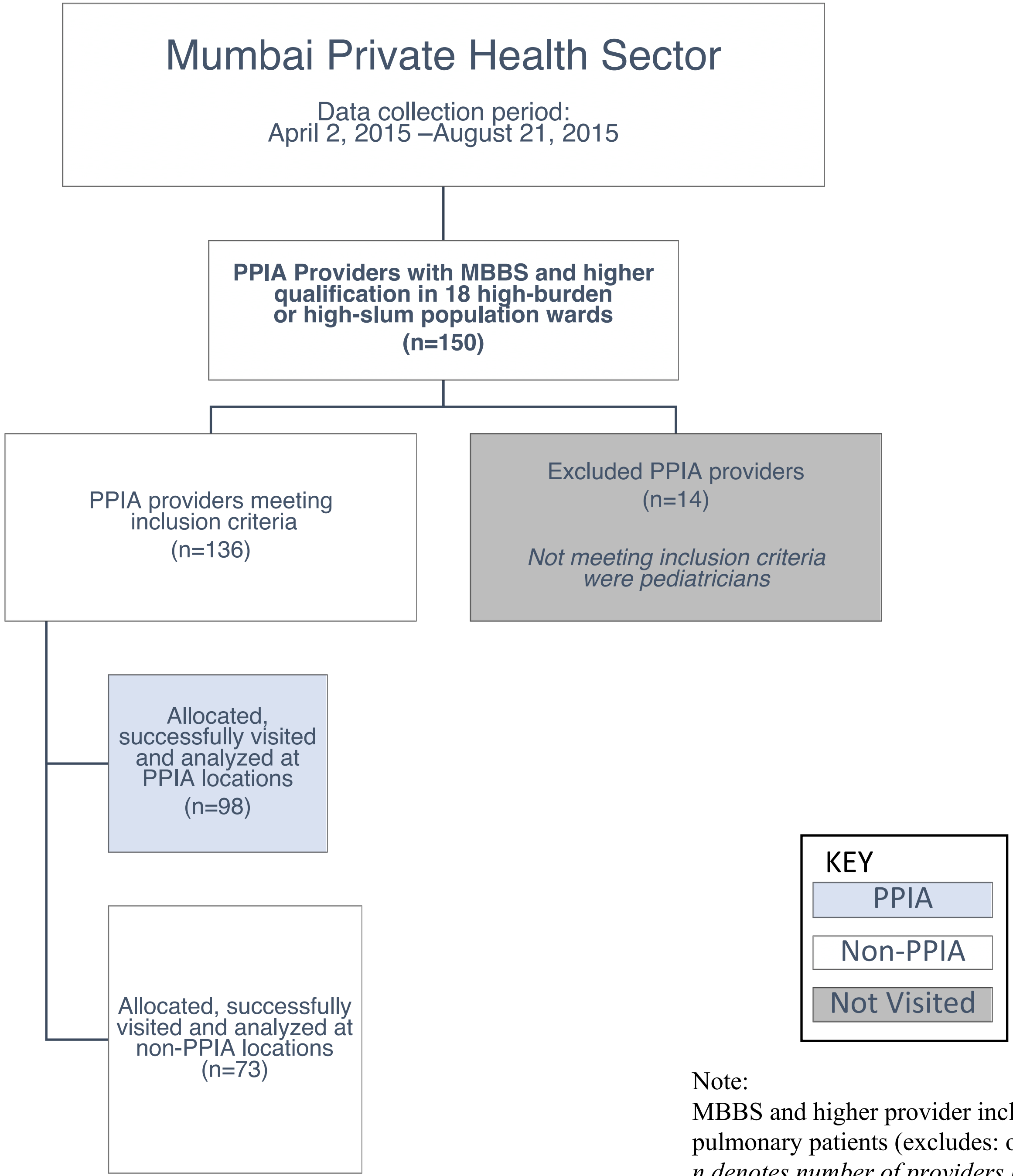

Note:
MBBS and higher provider inclusion criteria: providers who see adult pulmonary patients (excludes: orthopedists, pediatricians)
n denotes number of providers (overlaps with hub-level interactions)

Figure A2. Standard error simulations for SP-characteristic regressions

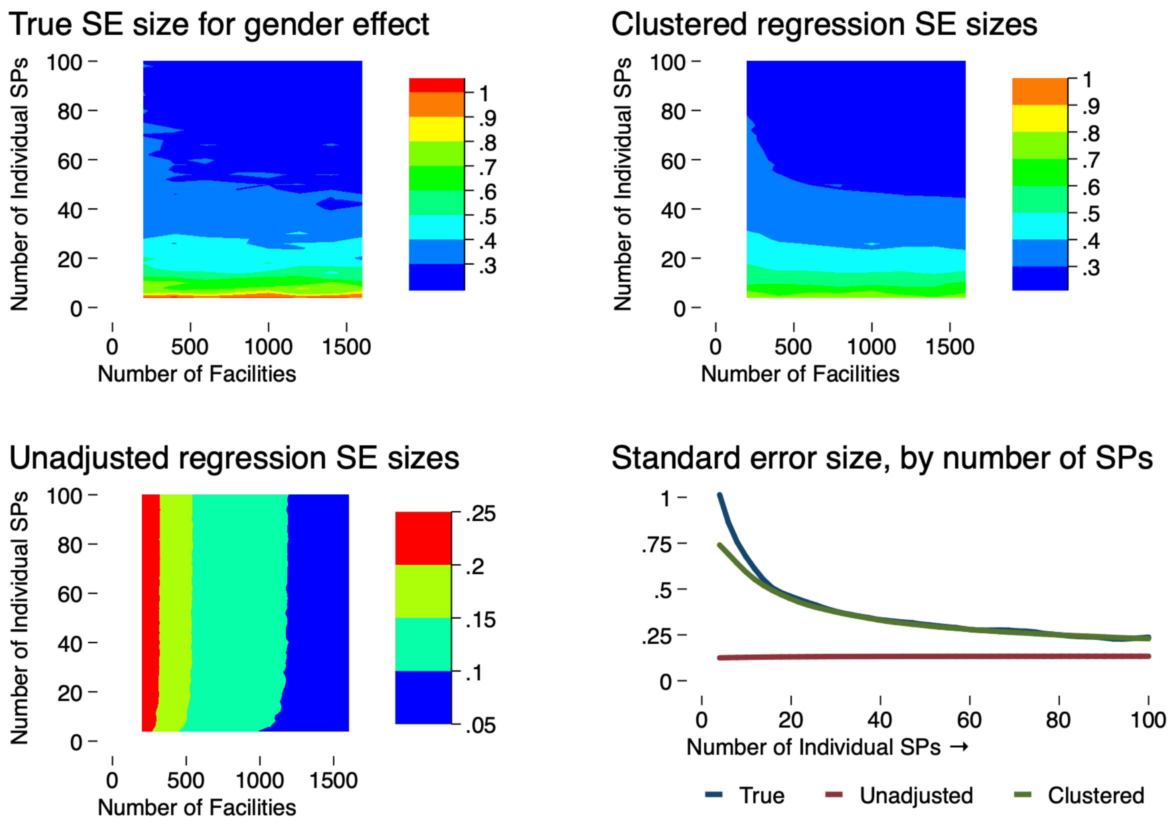

Notes: The upper-left panel of this figure illustrates the true way in which the point estimate of a single fixed parameter (in this case, SP gender) is subject to sampling error as the sample sizes for health care facilities and individual SPs change. The lower-left panel illustrates how ordinary, unadjusted regression estimates report that the sampling noise is reduced by increasing the number of facilities. The upper-right panel illustrates how the typical cluster-robust standard error adjustment estimates the noise in the parameter estimate. Finally, the lower-right panel illustrates how these three measures compare as the number of individual SPs increases.

Table A1. Standardized patient case management outcomes, by city and gender

	(1)	(2)	(3)	(4)
	Mumbai Female	Mumbai Male	Patna Female	Patna Male
**Correct Management**	0.47	0.33	0.32	0.33
	[0.38–0.55]	[0.29–0.37]	[0.26–0.38]	[0.28–0.38]
**Referred Case**	0.15	0.15	0.11	0.08
	[0.09–0.22]	[0.12–0.18]	[0.06–0.16]	[0.05–0.12]
**TB Suspicion**	0.61	0.55	0.40	0.37
	[0.53–0.69]	[0.51–0.59]	[0.33–0.47]	[0.32–0.43]
**Chest X-Ray**	0.43	0.42	0.38	0.45
	[0.34–0.51]	[0.38–0.47]	[0.32–0.44]	[0.4–0.51]
**Sputum AFB**	0.25	0.16	0.20	0.15
	[0.18–0.33]	[0.13–0.2]	[0.15–0.25]	[0.11–0.19]
**GeneXpert**	0.07	0.03	0.01	0.00
	[0.03–0.12]	[0.01–0.04]	[0–0.02]	[0–0.01]
**Any Medicine**	0.78	0.76	0.80	0.84
	[0.7–0.85]	[0.71–0.8]	[0.75–0.85]	[0.79–0.88]
**Anti-TB Medicine**	0.11	0.04	0.05	0.04
	[0.06–0.16]	[0.02–0.06]	[0.02–0.08]	[0.02–0.05]
**Fluoroquinolone**	0.09	0.08	0.22	0.21
	[0.03–0.14]	[0.05–0.11]	[0.16–0.27]	[0.17–0.26]
**Other Antibiotic**	0.42	0.36	0.40	0.41
	[0.33–0.5]	[0.32–0.41]	[0.33–0.46]	[0.35–0.46]
**Steroids**	0.11	0.15	0.09	0.11
	[0.07–0.15]	[0.13–0.17]	[0.05–0.13]	[0.06–0.15]
**N**	290	1,293	412	607

This table reports fully disaggregated outcomes by city and gender, for each of the case management behaviors with gender differences estimated in Figure 1. 95\% confidence intervals are reported in brackets below the means.

Table A2. Differences in exams and history taking by SP gender

	(1) N	(2) Mean	(3) N	(4) Mean	(5)
	Women		Men		Difference
**All Examinations**					
Pulse	702	0.52	1900	0.47	-.08
Auscultation	702	0.73	1895	0.81	.04
Temperature	702	0.21	1899	0.17	-.06
Throat Exam	702	0.35	1900	0.38	.02
Blood Pressure	702	0.48	1900	0.32	-.07**
Weight	702	0.28	1898	0.2	0
Abdomen palpitation	289	0.23	1293	0.24	.07*
**Case 1**					
Duration of Cough	268	0.96	1109	0.93	-.03
Sputum	268	0.84	1109	0.72	-.16***
Past TB	268	0.17	1109	0.16	-.02
Family TB	268	0.19	1109	0.11	-.07*
Blood in Sputum	268	0.3	1109	0.23	-.04
Cough Throughout Day	268	0.53	1109	0.4	-.11
Fever	268	0.82	1109	0.83	.04
Fever Type	268	0.6	1109	0.59	.02
Family Symptoms	268	0.12	1109	0.1	-.04**
Chest Pain	268	0.52	1109	0.25	-.27***
Loss of Appetite	268	0.49	1108	0.31	-.16***
Lost Weight	268	0.26	1109	0.18	-.11***
Wheezing	268	0.1	1109	0.04	-.08
Difficulty Breathing	268	0.41	1109	0.22	-.16***
Smoking	268	0	1109	0.23	.17***
Alcohol History	267	0	1109	0.09	.1***
Taken Medicines for Illness	268	0.69	1109	0.57	-.14**
Diabetes	268	0.12	1108	0.04	-.06
HIV/AIDS	268	0	1109	0.01	0
Age	268	0.76	1109	0.51	-.08
Provider Recorded Information	268	0.11	1108	0.09	-.03
**Case 2**					
Duration of Cough	196	0.93	189	0.95	.03
Sputum	196	0.56	189	0.67	.17***
Type of Doctor SP Saw	196	0.15	189	0.38	.11
Medicine Taken	196	0.3	189	0.37	.01
For How Long Medicines Were Taken	196	0.1	189	0.13	.01
Family TB	196	0.21	189	0.35	.12
Provider Saw X-Ray Film	196	0.89	189	0.86	-.1
Provider Read X-Ray Report	196	0.88	189	0.87	-.09
Blood in Sputum	196	0.2	189	0.26	.14***
Cough Throughout Day	196	0.11	189	0.41	.36**
Fever	196	0.68	189	0.72	.06
Fever type	196	0.5	189	0.48	-.03
Previous Typhoid Diagnosis	196	0.01	189	0.02	.01

Past TB	196	0.26	189	0.4	.13**
Family Symptoms	196	0.05	189	0.26	.17
Chest Pain	196	0.15	189	0.1	-.08*
Loss of Appetite	196	0.38	189	0.59	.16
Lost Weight	196	0.33	189	0.44	.12*
Wheezing	196	0.01	189	0	-.01
Difficulty Breathing	196	0.19	189	0.15	-.06
Smoking	196	0.01	189	0.29	.24**
Alcohol History	196	0	189	0.24	.18**
Taken Medicines for Illness	196	0.14	189	0.16	.05*
Diabetes	196	0.15	189	0.11	-.03*
HIV/AIDS	196	0	189	0.07	.06
Other Recent Illnesses	196	0.07	189	0.15	-.02
Age	196	0.62	189	0.58	-.01
Provider Recorded Information	196	0.07	188	0.15	.06
**Case 3**					
Duration of Cough	106	0.78	248	0.88	.1
Sputum	106	0.52	248	0.72	.18**
Saw Sputum Results	106	0.71	248	0.84	.09
Past Treatment for TB	106	0.13	247	0.34	.17**
Blood in Sputum	106	0.31	248	0.35	.03
Cough Throughout Day	106	0.43	248	0.52	.09*
Fever	106	0.81	248	0.86	.06
Fever Type	106	0.64	248	0.61	-.04
Family TB	106	0.25	248	0.18	-.13***
Family or Family with Similar Symptoms	106	0.15	248	0.1	-.09**
Chest Pain	106	0.14	248	0.22	.12**
Loss of Appetite	106	0.3	248	0.47	.21***
Lost Weight	106	0.25	248	0.33	.09
Wheezing	106	0.03	248	0.02	-.01
Difficulty Breathing	106	0.13	248	0.19	.06
Smoking	106	0	248	0.22	.12**
Alcohol	106	0.01	248	0.15	.03**
Taken Medicines for Illness	106	0.47	248	0.44	-.14**
Diabetes	106	0.02	248	0	-.01**
HIV/AIDS	106	0.01	248	0.01	-.02*
Children in Family	105	0.21	248	0.07	-.16***
Age	105	0.64	248	0.46	-.04
Provider Recorded Information	106	0.1	248	0.1	.01
**Case 4**					
Duration of Cough	132	0.95	354	0.95	0
Sputum	132	0.8	354	0.8	-.03
Saw Sputum Results	132	0.36	354	0.03	-.32*
Medication Taken Last Month for Present Illness	132	0.52	354	0.58	-.15
Visited Governmental Hospital for Previous Illness	132	0.37	354	0.15	-.29*
Treatment for Previous Illness	132	0.45	353	0.17	-.32
Sputum or X-Rays Done for Previous Illness	132	0.41	354	0.23	-.2
Diagnosis Given by Governmental Hospital	132	0.33	354	0.13	-.28*

Past TB Treatment	132	0.56	354	0.31	-.36
For How Long Past TB Treatment	132	0.75	354	0.66	-.05
Reason for Stopping	132	0.62	354	0.56	0
Previous Treatment Medical Records	132	0.58	354	0.34	-.15
Blood in Sputum	132	0.4	354	0.25	-.14**
Cough Throughout Day	132	0.58	354	0.51	-.13
Fever	131	0.74	354	0.77	-.07
Fever Type	132	0.45	354	0.49	-.03
Similar Symptoms Before	132	0.23	354	0.11	-.13
Family TB	132	0.27	354	0.14	-.16
Chest Pain	132	0.48	354	0.36	-.19*
Loss of Appetite	132	0.68	353	0.41	-.27***
Lost Weight	132	0.52	353	0.27	-.16*
Wheezing	132	0.17	353	0.04	-.15
Difficulty in Breathing	132	0.36	353	0.34	.02
Smoking	132	0.02	353	0.28	.2*
Alcohol	132	0.02	353	0.13	.1
Diabetes	132	0.1	353	0.08	-.05
HIV/AIDS	132	0.03	353	0.01	-.01
High Blood Pressure or Hypertension	132	0.18	353	0.12	-.1
Presence of Children	132	0.48	353	0.14	-.37*
Age	132	0.8	353	0.59	-.12*
Provider Recorded Information	132	0.14	353	0.14	.01

Table A3. Recent audit study designs, including number of locations and testers

Citation	Unit	Treatment	Sites	Testers	Location	Outcome	Type	Cites
1. Wright et al. (2013)	Employers	Religious affiliation	1,600	4	New England	Callback	Correspondence	1
2. Tilcsik (2011)	Employers	Sexual orientation	1,796	2	Seven states	Invitation to interview	Correspondence	49
3. Pager, Western, and Bonikowski (2009)	Employers	Felony, race	171/169	3	New York	Callback/offer	Audit	237
4. Drydakis (2009)	Employers	Sexual orientation	1,714	2	Athens, Greece	Callback, wage	Correspondence	64
5. Lahey (2008)	Employers	Age	3,996	2	Boston, St. Petersburg	Positive response, interview	Correspondence	101
6. Correll et al. (2007)	Employers	Parenthood	638	2	Northeast City	Callback	Correspondence	584
7. Bertrand and Mullainathan (2004)	Employers	Race, skill	1323	4	Chicago, Boston	Callback	Correspondence	1,664
8. Pager (2003)	Employers	Felony	350	2	Milwaukee	Callback	Audit	1,069
9. Weichselbaumer (2003)	Employers	Sexual orientation	613	2	Austria	Invitation	Correspondence	143
10. Bendick, Brown, and Wall (1999)	Employers	Age	102	2	Washington, DC	Favorable response	Audit	92
11. Bendick, Jackson, and Romero (1997)	Employers	Age	775	2	United States	Positive response	Correspondence	57
12. Esmail and Everington (1997)	Medical schools	Ethnicity	50	2	Great Britain	Callback, shortlist	Correspondence	47
13. Neumark, Bank, and Van Nort (1996)	Employer	Gender	65	2	Philadelphia	Callback	Audit	355
14. Ayres and Siegelman (1995)	Car dealers	Race, gender	153	2	Chicago	Offer price; accept counter	Audit	452
15. Bendick, Jackson, and Reinoso (1994)	Employers	Race	149	2	Washington, DC	Job offer	Audit	154
16. Kenney and Wissoker (1994)	Employer	Ethnicity	302	2	Chicago, San Diego	Callback, 3 levels	Audit	121
17. Esmail and Everington (1993)	Medical schools	Ethnicity	23	2	Great Britain	Callback, shortlisted	Correspondence	153
18. Turner and Mikelsons (1992)	Housing	Race	1,081/1,076/ 801/787	2	25 U.S. Metro Areas	3 Stages	Audit	27
19. Turner, Fix, and Struyk (1991)	Employers	Race	418	2	Chicago; Washington, DC	Job offer	Audit	218
20. Ayres (1991)	Car dealers	Race, gender	90	2	Chicago	Offer price; accept counter	Audit	645
21. Bendick et al. (1991)	Employers	Race	468	2	Washington, DC	Positive response	Correspondence	47
22. Riach and Rich (1991)	Employer	Race, ethnicity	519/462	2	Victoria, Australia	Interview offer	Correspondence	82
23. Yinger (1991)	Housing	Race	1,081/1,076/ 801/787	2	25 US Metros	3 stages	Audit	26
24. Riach and Rich (1987)	Employer	Gender	991	2	Victoria, Australia	Interview offer	Correspondence	50
25. Yinger (1986)	Housing	Race	156	2	Boston	Units offered	Audit	369
26. Feins and Bratt (1983)	Housing	Race	274	2	Boston	Available units	Audit	34

Note: reproduced from: Vuolo M, Uggen C, Lageson S. Statistical power in experimental audit studies: Cautions and calculations for matched tests with nominal outcomes. Sociological Methods & Research. 2016 May;45(2):260-303.
